# Reverse-bias enabled mesoscale shunt passivation for organic photovoltaic modules to power miniaturised Ambient IoTs under low-light conditions

**DOI:** 10.1038/s41467-026-72623-1

**Published:** 2026-05-06

**Authors:** Luhang Xu, Yuang Fu, Mianxin Xiao, Ho Ming Ng, Wenzhi Ma, Jun Yan, He Yan, Xin Li, Wei-Hsin Liao, Xinhui Lu

**Affiliations:** 1 Department of Physics, The Chinese University of Hong Kong, Shatin, Hong Kong China; 2 Department of Mechanical and Automation Engineering, The Chinese University of Hong Kong, Shatin, Hong Kong China; 3 Department of Chemistry and Hong Kong Branch of Chinese National Engineering Research Centre for Tissue Restoration and Reconstruction, The Hong Kong University of Science and Technology, Kowloon, Hong Kong China; 4 Guangdong Basic Research Centre of Excellence for Aggregate Science, School of Science and Engineering, The Chinese University of Hong Kong (Shenzhen), Shenzhen, Guangdong China; 5 School of Civil Engineering, Harbin Institute of Technology, Harbin, Heilongjiang China; 6 Institute of Intelligent Design and Manufacturing, The Chinese University of Hong Kong, Shatin, Hong Kong China

**Keywords:** Design, synthesis and processing, Electronic and spintronic devices

## Abstract

Organic photovoltaics (OPVs), with their intrinsic lightweight nature, flexibility, and low energy payback time, are promising power sources for Ambient Internet of Things (A-IoT) nodes. Yet, the large variation in shunt resistance compromises OPV reproducibility, especially for OPV modules operating under low-light environments, which are the typical working conditions of A-IoT nodes. This study reveals that random presence of mesoscale non-fullerene acceptor agglomeration is the primary contributor to leakage current in high-performance OPVs and demonstrates an effective shunt passivation method by applying a large, continuous reverse bias (RB) on as-fabricated devices. OPVs exhibit excellent stability during RB treatment, with leakage current flowing preferentially through shunted regions to generate spatially confined Joule heat, thereby promoting local molecular diffusion to selectively cure mesoscale shunt pathways. The RB-treated module, with an effective area of only 0.24 cm^2^, enables the continuous operation of our self-designed A-IoT temperature sensor under a minimal illuminance of 200 lux, representing the smallest self-powered A-IoT node operating under extremely low-light conditions. Our work presents a universally applicable method to overcome the key practical limitation in OPV module reliability, paving the way towards miniaturised, self-powered A-IoT nodes.

## Abstract (web summary)

Shunt resistance variation limits organic photovoltaic (OPV) module performance. Here, the author identified mesoscale non-fullerene acceptor agglomerations as major shunt pathways that can be passivated by reverse bias, enabling the smallest OPV-powered IoT nodes to operate at 200 lux.

## Introduction

Ambient Internet of Things (A-IoT) refers to an interconnected ecosystem of devices seamlessly integrated into environments that continuously provide data for analysis and decision-making to enhance daily life. Each individual device (referred to as an A-IoT node) is sustainably powered by ambient energy-harvesting technologies, among which photovoltaic (PV) is a widely adopted, technologically mature approach. Given that ambient lighting is often low in intensity and subject to temporal fluctuations (within the 200–1000 lux range), PVs must deliver superior performance in terms of efficiency, reliability, and deployability to meet stringent Quality of Service requirements^1–3^. In this context, organic photovoltaic (OPV) emerges as a promising third-generation PV technology, utilising non-toxic organic molecules as light absorbers and offering intrinsic lightweight properties, flexibility, and a low energy payback time^1,3–5^. While power conversion efficiencies (*PCEs*) of single-junction OPVs have surpassed 20% under outdoor light conditions and 30% under indoor light conditions, they still suffer from poor reproducibility (wide performance statistics) due to significant variations in shunt resistance (*R*_*sh*_) in as-fabricated devices^6,7^. A low *R*_*sh*_ compromises the open-circuit voltage (*V*_*OC*_) and fill factor (*FF*) of an OPV device, particularly under low-light indoor conditions where the magnitude of photocurrent becomes comparable to the leakage current^8^. For commercial modules with an increasing number of serially connected subcells, the random presence of shunted subcells further degrades the module’s performance under varying light intensities^9^. Consequently, large-area OPV modules are often employed to ensure sufficient power output, which in turn impedes the downsizing of A-IoT nodes for better deployability^3,10–12^.

The shunting of OPV is a two-step process involving the thermally activated injection of charge carriers from electrodes, followed by subsequent charge transport through the active layer^13^. Interfacial-induced shunting has been extensively investigated in early-generation ITO-free OPV devices incorporating heavily-doped PEDOT:PSS layers as electrodes. Due to the lack of charge carrier selectivity at the active layer:PEDOT:PSS (anode) interface, those devices exhibit initial ohmic shunting. This can be cured by applying a short reverse-bias pulse (10 ms) to induce electron accumulation, which de-dopes the PEDOT:PSS at the interface and forms a uniform electron-blocking layer, thereby improving the rectifying characteristics of the devices^14^. In high-performance OPVs, such interfacial-induced shunting has been eliminated through the employment of electron- and hole-transport layers (ETLs and HTLs) with excellent uniformity and charge carrier selectivity^13,15–18^. Therefore, recent works studying indoor OPVs primarily focused on passivating shunt pathways within the bulk of the active layer. This is usually achieved by optimising nanoscale phase-separated structures of the active layer through modifications to molecular structures, additive incorporation, and adjustments to post-annealing conditions^19–23^. However, since nanoscale morphology is also highly relevant to the generation and extraction of photo-generated charge carriers, those studies often result in case-specific conclusions that lack universal applicability^24^. On the other hand, the presence of mesoscale shunt pathways, which have been extensively studied for inorganic PVs, remains overlooked for OPVs^25,26^.

In this work, we revealed for the first time the presence of mesoscale shunt pathways in high-performance OPV systems and developed a universally applicable method for shunt passivation. Impedance spectroscopy, along with thickness- and composition-dependent dark *J*-*V* measurements, confirmed that the magnitude of leakage current in the prototypical PM6:Y6-based OPVs is determined by bulk-limited, filamentary-type charge transport through Y6-rich phases. Upon applying a large and continuous reverse bias (referred to as the RB treatment), we observed significant improvements in *R*_*sh*_ and device reproducibility. Conductive-atomic force microscopy (c-AFM) mappings revealed mesoscale Y6-rich agglomerates exceeding 1 μm in size within shunted devices. During the RB treatment, leakage current preferentially flows through these shunted regions, inducing spatially confined Joule heat that facilitates the local diffusion of Y6 molecules, thereby restoring mesoscale homogeneity. In the meantime, nanoscale phase separation and molecular orientation remain unmodified. The general applicability of our RB treatment is further confirmed across a variety of OPV systems utilising different active and charge-transport layer materials, in both normal and inverted structures. Encouragingly, the RB treatment is particularly effective at curing shunted subcells within OPV modules. With an effective area of only 0.24 cm^2^, our RB-treated OPV module powers our self-designed A-IoT temperature sensor under a minimal illuminance of 200 lux, representing the smallest self-powered A-IoT node operating under extremely low-light conditions. Our work significantly enhances the reliability of OPV devices, particularly in improving module performance under low-light conditions, thereby showcasing their potential as power sources in miniaturised, self-powered A-IoT nodes.

## Results

**Fig. 1:**
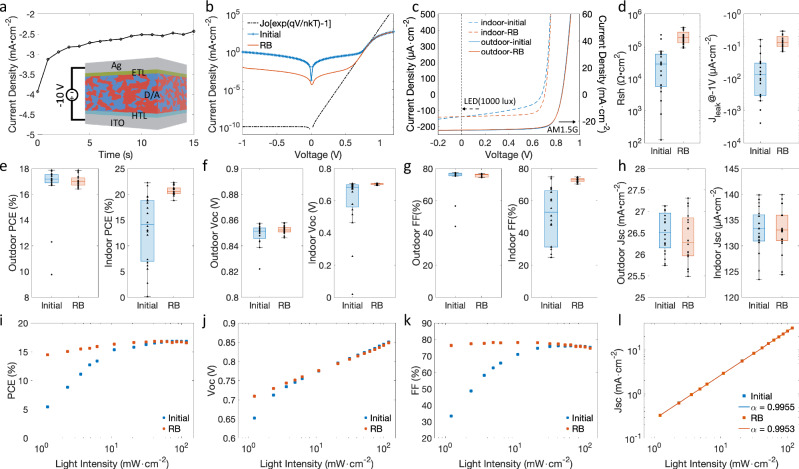
Photovoltaic performance of OPV devices before and after the RB treatment. **a** The real-time dark current density extracted during the RB treatment for 15 s. The setup of the RB treatment is shown in the inset, with −10 V bias applied to the ITO electrode (anode). **b** Dark *J-V* curves, **c** indoor (dashed lines) and outdoor (solid lines) light *J-V* curves of the same device before (blue lines) and after the RB treatment (orange lines). Statistical data of devices from the same batch: **d** *R*_*sh*_ and *J*_*leak*_, **e** *PCE*, **f** *V*_*OC*_, **g** *FF*, and **h** *J*_*SC*_ under outdoor and indoor light conditions. Light intensity-dependent performance of the same device before and after the RB treatment: **i** *PCE*, **j** *V*_*OC*_, **k** *FF*, and **l** *J*_*SC*_. The power-exponent α is shown in the inset of **l**.

We began with a prototypical OPV system employing a 100 nm blend film of polymer donor PM6 and small-molecule non-fullerene acceptor (NFA) Y6 as the active layer sandwiched within a conventional device structure of ITO/PEDOT:PSS/active layer/PNDIT-F3N/Ag (details of device fabrication are provided in the **Methods**). As illustrated in Fig. 1a, the reverse bias (RB) treatment involves applying a −10 V bias to the ITO anode (relative to the Ag cathode) in as-fabricated devices under dark conditions for 15 seconds, during which the dark current density undergoes a fast initial drop followed by saturation. Despite the thin active layer, OPVs appear robust during RB treatment with negligible degradation in device performance after prolonging the duration to 1 hour (Fig. S1). This is in stark contrast to perovskite PVs, which show semi-irreversible degradation even under mild reverse bias^27,28^. The magnitude of voltage used in the RB treatment (−10 V) has been optimised to maximise the curing effect while reducing the risk of device breakdown, as discussed in detail in Figs. S2–4. In contrast, Figure S5 demonstrates that shunt passivation cannot be achieved under forward bias (FB) treatment; instead, large FB degrades the device performance.

As shown in Figs. 1b, c, S6, dark and light *J-V* curves were measured for the same batch of 20 devices under outdoor (AM1.5 G) and indoor (2600 K LED with an illuminance of 1000 lux) light conditions before and after the RB treatment, labelled as ‘initial’ and ‘RB’, respectively. The dark current density (*J*_*d*_) in an OPV device is composed of dark saturation current density (*J*_*0*_) and leakage current density (*J*_*leak*_)^13^. By fitting the exponential part of the dark *J*-*V* curve using the Shockley equation (Fig. 1b), we obtained the *J*_*0*_ on the order of 10^−10 ^mA cm^−2^ and confirmed that *J*_*leak*_ is indeed the main contributor to *J*_*d*_, typical for OPVs with thin active layers. Therefore, we used *J*_*d*_ measured at −1 V to estimate *J*_*leak*_ and used the inverse differential of the dark *J*-*V* curve at 0 V to calculate *R*_*sh*_. As shown in the statistical data (Fig. 1d–h, Table S1), initial devices suffer from a large scatter in the magnitudes of *R*_*sh*_ and *J*_*leak*_, which compromises device reproducibility primarily by influencing *FF* and *V*_*OC*_. Encouragingly, the *J*_*d*_ of the RB-treated device is reduced by two orders of magnitude (Fig. 1b), while light *J-V* curves become much more square-like with enhanced *FF* and *V*_*OC*_ (Figs. 1c, S6). Benefitting from the higher *R*_*sh*_ and suppressed *J*_*leak*_, RB-treated devices exhibit better average performance with a much narrower distribution than initial devices (Fig. 1e). Compared to outdoor light conditions, the effect of the RB treatment is more pronounced under indoor light conditions (Fig. 1c), where *J*_*leak*_ has a greater effect on device performance due to the much lower photocurrent.

To further understand the roles of shunt pathways on key device metrics, we performed light-intensity (*I*)-dependent and temperature-dependent (*T*)-dependent *J*-*V* measurements on the same device before and after the RB treatment, as shown in Figs. 1i–l, S7. *FF* and *V*_*OC*_ drop significantly with decreasing light intensity in the initial device, as previously explained using a simple equivalent circuit model incorporating a finite shunt resistance^8^. In contrast, the *V*_*OC*_ of the RB-treated device strictly follows the ideal *kT/q*ln(*I*) dependence throughout the measured intensity range, while *FF* remains almost constant due to effective shunt passivation. On the other hand, the intensity-dependent *J*_*SC*_ curve shows no discernible difference before and after the RB treatment, consistent with the device statistics (Fig. 1h) and results of external quantum efficiency (*EQE*) measurements (Fig. S8). This is also consistent with our equivalent circuit modelling results, which indicate that *J*_*SC*_ is barely influenced by *R*_*sh*_ when it is larger than 100 Ω⋅cm^2^ (Fig. S9). Recent work has also suggested that high leakage current influences temperature-dependent *V*_*OC*_ measurements, causing an anomalous turnover at low temperatures^29^. Encouragingly, we found that the RB treatment can also cure such behaviour, restoring the expected near-linear *V*_*OC*_ increase with decreasing temperature (Fig. S7). Overall, the alignment between device statistics, light-intensity-dependent, and temperature-dependent measurements clearly demonstrates the effectiveness of our RB treatment in enhancing the performance and reproducibility of OPV devices, particularly under low-light conditions.

**Fig. 2:**
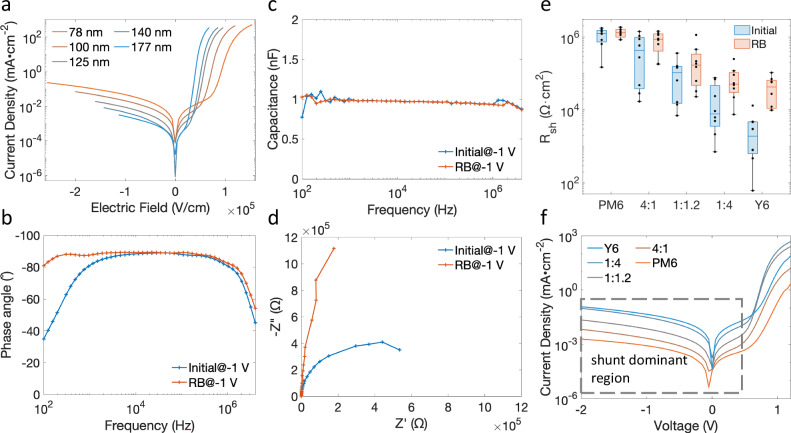
The conduction mechanism of leakage current. **a** *J*_*d*_ of RB-treated devices with different active layer thicknesses plotted against the electric field. **b** Bode (phase) plots, **c** capacitance spectra, and **d** Nyquist plots of initial and RB-treated devices at a DC bias of −1 V. **e** Statistics of *R*_*sh*_ for initial and RB-treated devices with different D/A ratios. **f** Dark *J-V* curves of typical devices with different D/A ratios.

Next, we elucidated the conduction mechanism of *J*_*leak*_ in our devices via thickness-dependent dark *J*-*V* measurements. By plotting dark current density against the electric field (Fig. 2a), we observed a gradual reduction in *J*_*leak*_ with increasing active layer thickness. This excludes the possibility of injection-limited *J*_*leak*_, which is expected to be independent of active layer thickness under the same electric field^30^. To understand the nature of this bulk-limited conduction mechanism, impedance spectroscopy measurements were conducted at a DC bias of –1 V under dark conditions. As shown in Fig. 2b, at frequencies above 10^4 ^Hz, where the impedance is dominated by capacitive response, the Bode (phase) plots of both the initial and RB-treated devices merge. This is consistent with the capacitance spectra of initial and RB-treated devices, which show identical magnitudes and slopes within the measured frequency range (Fig. 2c), ruling out the potential contribution of bulk traps to *J*_*leak*_^31,32^. At frequencies below 10^4 ^Hz, where the impedance is dominated by *R*_*sh*_, the phase angle of the RB-treated device remains at around −90° while that of the initial device shows significant deviation due to insufficient *R*_*sh*_. Consistently, the initial device shows a much smaller semicircle radius compared to the RB-treated device in Nyquist plots (Fig. 2d). Those results point out that *J*_*leak*_ in PM6:Y6 devices is dominated by bulk-limited charge transport at local shunted regions of the active layer, also known as filamentary-type conduction^31^. To determine the composition of conductive filaments, we systematically varied the D:A ratios of the active layer while maintaining a constant thickness of 100 nm to monitor the change in *J*_*leak*_^33^. Fig. 2e, f demonstrate that the increase of Y6 content led to a reduction of *R*_*sh*_ and an increase of *J*_*leak*_ for both initial and RB-treated devices. This indicates that the Y6-rich phase functions as local conductive filaments for *J*_*leak*_.

**Fig. 3:**
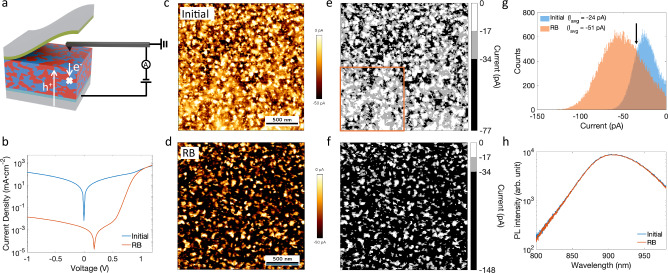
The morphological origin of the shunt pathways. **a** Setup of c-AFM measurement. The positive bias was applied to the substrate for hole injection, while the grounded probe was placed on the top surface of the active layer after peeling off the top electrode and ETL. **b** Dark *J-V* curves of PM6:Y6 (CB) devices without and with the RB treatment for c-AFM measurements. The current mappings (**c**) without and (**d**) with the RB treatment. **e** and **f** The re-colorized c-AFM mappings corresponding to **c** and **d**. The orange box in (**e**) with a size of 1 µm×1 µm is used to highlight the presence of mesoscale Y6-rich agglomerates. **g** The current histograms of two c-AFM mappings. The corresponding micro-PL spectra are shown in (**h**).

To investigate the curing effect of the RB treatment on Y6-rich conductive filaments, c-AFM measurements were performed on initial and RB-treated devices after peeling off the top electrode and electron transport layer (ETL) using tape, thereby exposing the top surface of the active layer, as shown in Fig. 3a. The PM6:Y6 device processed with chlorobenzene (CB) was studied first (dark *J*-*V* curves shown in Fig. 3b and photovoltaic performance shown in Fig. S10, Table S2), as this system exhibits stronger phase segregation, allowing more confident assignments of PM6- and Y6-rich phases^34^. By applying a positive bias to the PEDOT:PSS/ITO substrate for hole injection, and using a grounded c-AFM tip to collect holes that reach the top surface, our c-AFM measurement probes the difference in the 3-D hole transport network within the bulk active layer^35^. Considering interfacial energetic alignment (Fig. S11**)**, holes can be effectively injected from the ITO/PEDOT:PSS substrate to PM6-rich phases due to its shallower highest occupied molecular orbital (HOMO), while the injection current falls significantly in the Y6-rich phases with a much deeper HOMO, as confirmed by c-AFM mappings of pure PM6 and Y6 films (Fig. S12). Therefore, the increased magnitude of (negative) hole current upon the RB treatment, as shown in Fig. 3g, indicates the formation of more interconnected PM6-rich hole-transport pathways. To better visualise the morphology change, we re-rendered the c-AFM mappings (Fig. 3c, d) using a three-colour scale (Fig. 3e, f). As shown in Fig. 3c, d, c-AFM mappings of both initial and RB-treated devices exhibit bright spherical regions with local currents between 0 and −17 pA. Those regions, rendered white, are assigned to Y6 crystalline domains with a size of around 50 nm (Fig. S13), consistent with our previous works^34^. In the initial device, there are large, interconnected low-current regions with sizes exceeding 1μm surrounding Y6 crystalline domains. Those regions, with local current between −17 pA and −34 pA (the intersection point between the current histograms of the initial and the RB-treated devices), are assigned to the mesoscale Y6-rich agglomerates and rendered grey. At last, regions with the magnitudes of local current exceeding 34 pA, which appear predominantly in the RB-treated device, are rendered black. Those regions are assigned to homogenised mesoscale phases with more interconnected PM6-rich hole-transport pathways, thereby suppressing Y6-rich shunt pathways. Based on the re-rendered c-AFM mappings (Fig. 3e, f), it becomes clear that the main impact of the RB treatment is to annihilate mesoscale Y6-rich agglomerations that function as local shunt pathways. Consistent results were also obtained when the mapping area was increased from 2 × 2 µm^2^ to 5 × 5 µm^2^ (Fig. S14).

To distinguish our RB treatment from the well-known electrical annealing method, which affects nanoscale morphology and the molecular orientation, we measured micro-photoluminescence (PL) spectra on initial and RB-treated devices, which completely overlap with each other, as shown in Fig. 3h^36–41^. This is consistent with the results of *J*_*SC*_ statistics (Fig. S10d), light-intensity-dependent *J*_*SC*_ (Fig. S10i), and *EQE* measurements (Fig. S15), confirming that the nanoscale phase separation, which governs the yield of exciton dissociation and charge generation, is unmodified by RB treatment^24^. Additionally, the topography and surface potential (SP) mappings obtained by tapping-mode AFM and Kevlin Probe Force Microscopy (KPFM, Fig. S16) also show no discernible difference with and without the RB treatment. Due to the large molecular quadrupole moment associated with Y-series NFAs, any change in molecular orientation is expected to result in a notable change in the SP of the film^42^. Therefore, the identical SP indicates that the molecular orientation remains unchanged after the RB treatment. Finally, optical microscopy images of the initial and RB device appear identical and smooth (Fig. S17), which excludes the potential impact of macroscopic defects, such as pinholes, on *R*_*sh*_. Consistent results were also observed in high-performance CF-processed PM6:Y6 devices, as shown in Fig. S18–S21.

**Fig. 4:**
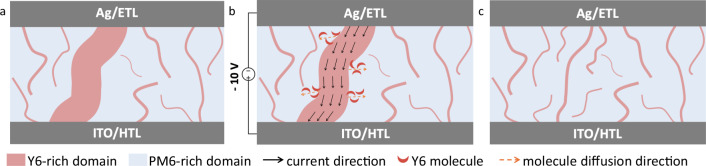
Annihilation of mesoscale shunt pathways in the OPV active layer. **a** The initial D/A network. **b** The molecule diffusion due to the thermal gradient during the RB treatment. **c** The D/A network after the RB treatment.

Based on the above results, we propose the fundamental mechanism of RB treatment, as illustrated in Fig. 4. The random presence of mesoscale Y6-rich agglomerates is the key contributor to the large variation of *R*_*sh*_ in initial devices. This likely arises from the much worse rectifying characteristic of Y6 than PM6 (Fig. 2e, f), due to the smaller bandgap of Y6 as well as its stronger tendency to aggregate (via face-on π-π stackings)^43^. During the RB treatment, large leakage current preferentially flows through those Y6-rich agglomerates, which generates spatially confined Joule heat to induce local diffusion of Y6 molecules, selectively annihilating those mesoscale shunted pathways. In the meantime, the nanoscale morphology and molecular orientation are unaffected by the RB treatment, maintaining efficient photocharge generation and extraction. In contrast, the joule heat generated under FB treatment is uniformly distributed across the entire active layer, which, as proposed by Maria et al., is equivalent to thermally annealing the device at the same temperature^41^. In fact, the excessive joule heat generated under the FB treatment, which is over five orders of magnitude higher than that under the RB treatment (as determined by comparing the magnitudes of current flow during the RB treatment at −5 V shown in Fig. S3b and the FB treatment at 5 V shown in Fig. S5a), can degrade the device performance. Therefore, the unique advantage of the RB treatment is that it leverages the rectifying characteristics of the diode structure, allowing leakage current to selectively anneal and cure the local shunted region.

To assess the general applicability of our RB treatment, we applied it to a wide range of NFA-OPV systems, with different active layer materials and charge transport layers, in both normal and inverted structures, as summarised in Fig. S22 and Table S3. Before the RB treatment, all devices showed scattered *R*_*sh*_ values, which resulted in large variations in indoor photovoltaic performance. For BTRCl:Y6, small shunt resistance readily harms outdoor performance, so the corresponding indoor performance was not measured. Promisingly, after RB treatment, all devices showed improved photovoltaic performances, along with much better statistics. The general applicability of the RB treatment on NFA-OPV systems with vastly different optoelectronic and morphological properties suggests that the presence of mesoscale inhomogeneity within the bulk active layer is a universal characteristic of solution-processed NFA-OPVs. Therefore, the RB treatment can be employed as a standard post-treatment method to improve the reproducibility of high-performance NFA-OPVs, especially for those designed for indoor applications.

**Fig. 5:**
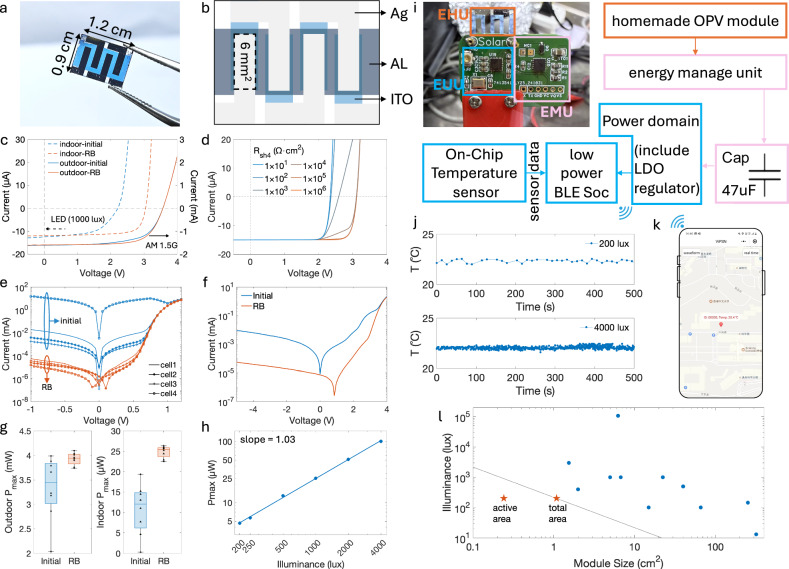
RB-treated OPV modules as the power sources for A-IoT nodes. **a**, **b** The photo and schematic of the OPV module. **c** Indoor (dashed lines) and outdoor (solid lines) light *J-V* curves before (blue lines) and after the RB treatment (orange lines). **d** The simulated *J-V* curves under light intensity of 1 mW⋅cm^−^^2^ using a four-diode model. Dark *J-V* curves of **e** each subcell and **f** the whole module before (blue lines) and after (orange lines) the RB treatment. **g** Statistical P_max_ of 8 independent modules before and after the RB treatment. **h** *P*_*max*_ of an RB-treated module plotted against the illuminance. **i** System-level block diagrams of the A-IoT temperature sensor integrated with an OPV module. **j** Real-time temperature monitoring under different illuminances. **k** The real-time transmission of temperature and location data to a smartphone. **l** A comparison of illuminance and the area of PV module used as power supply for A-IoT nodes in the last five years, with the demonstration in this work. Detailed parameters are included in Table S7. For fair comparison, we include both the total area of our module (1.08 cm^2^) and the active area (0.24 cm^2^). The solid line represents a constant illuminance–module size product, serving as a visual guideline.

To evaluate the effectiveness of our RB treatment at the module level, we implemented it in our custom-designed OPV module comprising four 0.06 cm^2^ subcells connected in series with a device structure of ITO/2PACZ/PM6:L8BO (100 nm)/PDINN/Ag, as shown in Fig. 5a, b. Under outdoor light conditions, the initial module shows compromised *FF* due to an ‘early turn-on’ in current below the built-in voltage, as shown in Fig. 5c (blue solid line) and Fig. S23a. Under indoor light conditions (Fig. 5c, blue dashed line), the initial module shows a more significant reduction in *PCE* due to an additional *V*_*OC*_ loss. To examine the origin of inferior module performance, we performed separate dark *J*-*V* measurements on each of the four subcells. As shown in Fig. 5e and Table S4, four subcells exhibit vastly different degrees of shunting with *R*_*sh*_ ranging from 4 Ω⋅cm^2^ to 5.4 × 10^5^ Ω⋅cm^2^. To understand the underlying mechanism, we simulated the module *J*-*V* curves under various light intensities using a four-diode model as shown in Fig. S24a. We set the *R*_*sh*_ of three diodes to 1 × 10^6^ Ω⋅cm^2^, which is close to the *R*_*sh*_ measured in RB-treated devices, and varied the *R*_*sh*_ of the fourth diode (*R*_*sh4*_) from 1 × 10^6^ to 10 Ω⋅cm^2^. As *R*_*sh4*_ decreases, the leakage current first influences the diode current near *V*_*OC*_, resulting in the “early turn-on” observed in the outdoor light *J*-*V* curve that compromises *FF* (Figs. 5d, S24b–e). Upon further decreasing light intensities and *R*_*sh4*_, the magnitude of photocurrent becomes comparable to the leakage current in the fourth diode, so it behaves like a resistor, giving rise to the additional *V*_*OC*_ loss observed under indoor light conditions. Therefore, our simulation suggested that the large variation of *R*_*sh*_ among subcells is the main cause of the compromised OPV module performance under outdoor and indoor light conditions. By applying the RB treatment to each subcell, their average *R*_*sh*_ was significantly increased to over 10^6^ Ω⋅cm^2^, and dark current density was decreased by several orders of magnitude (Fig. 5e, Table S4), leading to a largely suppressed dark current within the entire module, as shown in Fig. 5f. As a result, light *J*-*V* curves of the module become square-like under both outdoor and indoor conditions, with significant improvements in all photovoltaic parameters (Figs. 5g, S23, S25, Table S5), consistent with the simulation results. We further measured the performance of the RB-treated OPV module within a wide illuminance range from 4000 to 200 lux (Fig. S26, Table S5**)**. Remarkably, the maximum power output (*P*_*max*_) of the RB-treated module maintains a nearly linear proportionality against the illuminance (Fig. 5h), showcasing its excellent reliability.

To demonstrate real-world applicability, we employed our RB-treated OPV module as the power source in a miniaturised, self-powered A-IoT node. The power consumption of typical A-IoT nodes is on the mW-scale, often requiring panel-scale PV modules with an effective area of tens of cm^2^ (Table S7), which constrains downsizing and deployment^10,12,44^. Herein, we designed a BLE (Bluetooth Low Energy)-equipped temperature sensor with an ultra-low power consumption of only 4 µW. This is achieved by avoiding the use of high-consumption components, such as DC-DC converters or low-dropout (LDO) regulators, in our circuit design. Instead, we utilise the simplest dual Under-Voltage Lockout (UVLO) circuit to manage energy flow (Fig. S27), which fundamentally eliminates unnecessary circuit complexity and switching losses. The temperature sensor and an RB-treated OPV module with an active area of only 0.24 cm^2^ are integrated at a micro-patch scale (Fig. 5i), achieving self-powered operation within a wide illuminance range from 4000 to 200 lux (Fig. 5j, S28). During operation, data collected from the device is delivered via the BLE beacon to a smartphone and synchronised to the cloud (Fig. 5j, k). The minimal execution interval of around 200 ms can be achieved for illuminance above 250 lux, while the average execution interval decreases continuously with increasing illuminance, reaching 566 ms at 4000 lux (Fig. S29). Remarkably, under an extremely low illuminance of 200 lux, at which the *P*_*max*_ of our OPV module (4.62 µW, see Table S5) is close to the startup power of our A-IoT node (4 µW), the node still operates continuously with a decent average execution interval of 14 s and a minimal execution interval of 12 s, as shown in Table S6. By benchmarking our results with previous works (Fig. 5l), it becomes apparent that our prototype represents the smallest self-powered A-IoT node that can operate under extremely low-light conditions. The downsizing of A-IoT nodes, achieved through the joint efforts of our RB treatment and innovative circuit design, will enable the transition from panel-scale deployment to sticker-scale deployment, representing a crucial step towards further expanding the application scenarios of A-IoTs.

## Discussion

Overall, our work identifies mesoscale NFA agglomerations as the primary contributors to leakage currents in high‑performance OPVs. Such mesoscale inhomogeneity randomly presents within solution‑processed active layers, leading to scattered shunt resistance and compromised device reproducibility. This effect is particularly detrimental under low-light indoor environments, which are the typical working conditions for A-IoT nodes. To address this challenge, we developed a simple yet effective shunt-passivation method, known as the RB treatment. Despite the thin active layer, OPVs remain robust during RB treatment, as leakage current preferentially flows through shunted NFA filaments to generate spatially confined Joule heat, which induces local molecular diffusion to annihilate shunt pathways. Unlike conventional post‑treatments, RB treatment selectively cures mesoscale shunted regions without disturbing nanoscale morphology and molecular orientation, making it broadly compatible with diverse fabrication protocols. Since light-intensity-dependent and temperature-dependent *J*-*V* measurements that are widely applied to study the recombination kinetics and energetics of OPVs can be influenced by shunt pathways, the RB treatment is suggested to be performed before those measurements to eliminate the random shunting effect and thereby better reveal the intrinsic properties of the materials systems under investigation. Finally, the practical utility of RB treatment is further demonstrated by our OPV module with an effective area of only 0.24 cm^2^, which powers our self-designed BLE-equipped temperature sensor under an extremely low illuminance of 200 lux. By ensuring predictable power delivery at sub‑cm^2^ scale while sustaining standard‑protocol connectivity, our approach advances passive labels into active endpoints and transitions OPV-IoT from pilot‑scale demonstrations toward pervasive, sticker‑scale infrastructure.

## Methods

### Materials

Chloroform (CF), chlorobenzene (CB), 1-chloronapthalene (CN), Zn(CH_3_COO)_2_·2H_2_O, 2-Methoxyethanol, ethanolamine, and MoO_3_ were purchased from Sigma-Aldrich. 1,4-diiodobenzene (DIB) and 2PACz were purchased from Meryer Co., LTD. PM6 and Y6 were purchased from Solarmer Inc. (Beijing). BTRCl, L8BO, Y6-1O, PDINN, and PDINO were purchased from Derthon Optoelectronic Materials Science Technology Co., Ltd. PNDIT-F3N was purchased from eFlexPV Ltd. PEDOT:PSS (Al 4083) was purchased from Heraeus Ltd. All chemicals were used as received without further purification.

### Device fabrication and characterisation

For conventional devices and homemade modules, the organic photovoltaics (OPVs) were fabricated with the structure of ITO/hole transport layer (PEDOT:PSS or 2PACz)/active layer/electron transport layer (PNDIT-F3N, PDINN, or PDINO)/Ag (100 nm). The ITO substrates were sequentially ultrasonicated for 20 min using detergent, deionized water, acetone, and isopropanol, and then treated with UV-ozone for 20 min. PEDOT:PSS was spin-coated onto ITO at 4000 rpm for 30 s and dried at 120 ˚C for 20 min in ambient. 2PACz (0.3 mg/ml) in ethanol was spin-coated onto ITO at 3000 rpm for 30 s after 15 s resting and heated at 100 ˚ C for 10 min in the N_2_-filled glovebox. The material for the active layer was dissolved in different solvents. For PM6:Y6 and PM6:Y6-1O-based devices, materials were dissolved in CF with 0.5 vol% CN as an additive. For PM6:BTP-eC9, PM6:L8BO, and BTRCl:Y6-based devices, materials were dissolved in CF with 10 mg/ml DIB as an additive. For PM6:L8BO-based indoor devices, materials were dissolved in CF without an additive. For PM6:Y6 (in CB)-based devices, materials were dissolved in CB with 0.5 vol% CN as an additive. The D/A ratio is 1:1.2, except the BTRCl:Y6 (1.7:1). The active layer was spin-coated at 3000 rpm for 30 s and annealed at 100 ˚ C for 5 mins. The active layer, composed of PM6:L8BO without an additive, was annealed at 80 ˚ C for 5 minutes. PNDIT-F3N (0.5 mg/ml with 0.5 vol% ethanoic acid), PDINN (1 mg/ml), or PDINO (0.5 mg/ml) in methanol was deposited at 2000 rpm for 30 s. Then, the Ag electrode with a thickness of around 100 nm was thermally evaporated at 10^-4^ Pa.

For an inverted device, OPVs were fabricated with the structure of ITO/ZnO/active layer/MoO_3_(2.6 nm)/Ag (100 nm). The ZnO precursor was prepared by dissolving Zn(CH_3_COO)_2_·2H_2_O (100 mg) in 2-methoxyethanol (973 µL) with ethanolamine (28.29 µL). After fully mixing, the precursor was stirred at 60 ˚C for 10 min, followed by stirring at room temperature overnight. The ZnO layer was spin-coated onto ITO substrates at 4000 rpm for 30 s in air and annealed at 200 ˚ C for 30 min. The active layer was deposited as the conventional device. Then, the MoO_3_ layer and the Ag electrode were thermally evaporated at 10^−^^4^ Pa.

The current density-voltage (*J*-*V*) curves of devices were measured by a Keithley 2400 Source Metre in a N_2_-filled glove box under various light sources. For the outdoor condition, a solar simulator (SS-F5-3A, Enlitech) with AM 1.5G (100 mW⋅cm^−^^2^) spectrum was used, and the intensity was calibrated by a reference silicon solar cell (SRC2020, Enlitech). For the indoor condition, a white LED (iwata M1 Pro RGB Mini, iwata Tech) with adjustable colour temperature and intensity was used. The illuminance was calibrated by a light metre (TES-1334N, TES Electrical Corp.). The external quantum efficiency (EQE) was measured by a QE/IPCE system (Enli Technology Co. Ltd., China) in a wavelength range of 300−1000 nm. The thickness of the active layer was measured by a profilometer (Bruker Dektak XT).

### Temperature-dependent *J–V* measurement

Current density–voltage characteristics were measured using an automatic photovoltaic efficiency measurement system equipped with a commercial solar simulator (LIV-1220, LightSky Technology Co., Ltd.). For temperature-dependent measurements, the devices were mounted on a Linkam HFS600E-PB4 heating/freezing stage integrated into the optical path of the measurement system. The stage provides a temperature range from −196 to 600 °C with a temperature stability of 0.1 °C. During the measurements, the sample chamber was continuously purged with dry nitrogen to minimise moisture accumulation and ice condensation at low temperatures. At each target temperature, the stage was set to the desired value and held until thermal equilibrium was reached, as confirmed by a temperature fluctuation within ±0.1 °C, after which the *J–V* curve was recorded. All measurements were performed under a fixed one sun intensity condition.

### Impedance spectroscopy

The impedance spectroscopy was measured by ZAHNER ZENNIUM Electrochemical Workstation. All devices were encapsulated and measured in air. During the measurement, a 10 meV AC bias was applied to the device with frequency scanning from 4 MHz to 100 Hz at the reverse bias (−1.0 V) to obtain the complex impedance of the device under dark conditions. The capacitance spectrum was derived from the complex impedance using Eq. 1^32^:

1 $${C}_{{\mbox{cor}}}=-\frac{1}{\omega }\left[\frac{{Z}^{{\prime} {\prime} }-\omega L}{{\left({Z}^{{\prime} }-{R}_{{{\rm{s}}}}\right)}^{2}+{\left({Z}^{{\prime} {\prime} }-\omega L\right)}^{2}}\right]$$

where *Z’* and *Z”* are the real and imaginary parts of the complex impedance, *ω* is the angular frequency, *R*_*s*_ is the series resistance of the devices derived from the dark *J-V* curve, and *L* is the parasitic inductance.

### Atomic force microscopy

All AFM images were conducted using the JPK NanoWizard NanoOptics from Bruker. All samples were fabricated by peeling off the top electrode and electron transport layer using tape and measured in air. Topography images and surface potential mapping were taken through Klevin force probe microscopy on TappingMode^tm^ technology using the conductive ElectriMulti75-G probe (Pt overall coating, Budget Sensors) in the dark condition. c-AFM images were taken under the contact mode via the conductive ElectriCont-G probe (Pt overall coating, Budget Sensors). The 2 V bias was applied to ITO to inject holes into the active layer in the dark conditions.

### Micro-photoluminescence spectrum

The Renishaw inVia Qontor Micro Raman was used to measure PL spectroscopy with a 785 nm excitation laser. The sample was fabricated by peeling off the electron transport layer and Ag electrode to expose the surface of the active layer for measurement.

### Optical microscopy

The Olympus BX60 with AxioCam MRc 5 (ZEISS) and 10× objective lens (Olympus) was used to measure optical microscopy. The sample was fabricated by peeling off the electron transport layer and Ag electrode to expose the surface of the active layer for measurement.

### Module *J*-*V* curve simulation

The four-diode model was simulated module *J*-*V* curves using MATLAB Simulink. The solar cell block was parameterized by s/c current and o/c voltage, 5 parameters. A PS constant block was used to simulate the light source. A resistor block placed in parallel with the solar cell block was used to simulate the shunt resistance. A resistor block placed in series with the solar cell block was used to simulate the series resistance. A Piecewise Linear Voltage Source block was used to generate a voltage for the circuit from −5V to 5 V. A current sensor, a voltage sensor, and a scope were used to measure the voltage and current in the circuit.

### A-IoT sensor design

The A-IoT sensor circuit consists of three main components: the Energy Harvester Unit (EHU), the Energy Management Unit (EMU), and the Energy Utilization Unit (EUU). The EMU includes two capacitors for energy storage: a $1\,\mu {{\rm{F}}}$ capacitor, which ensures the normal operation of the energy management circuit, and a $47\,\mu {{\rm{F}}}$ capacitor, denoted as ${C}_{{{\rm{sto}}}}$, which serves as the primary energy reservoir for the EUU. The EMU also integrates a hysteresis comparator, a window comparator, and three switches that control the energy flow. The hysteresis comparator features high and low threshold voltages (${V}_{{{\rm{DET}}}}$) of 1.55 V and 1.45 V, respectively. When the voltage at the EHU input to the EMU reaches the upper threshold of the hysteresis comparator (1.55 V), switch SW2 (Fig. S27) is activated, establishing a direct connection between the EHU and the $47\,\mu {{\rm{F}}}$ storage capacitor. At this point, the $1\,\mu {{\rm{F}}}$ capacitor maintains the EHU voltage and continues to power the energy management circuit. Conversely, when the EHU voltage falls below the lower threshold of the hysteresis comparator (1.45 V), the internal control circuit deactivates SW2, preventing reverse discharge from the storage capacitor to the EHU, and ensuring readiness for the next power cycle. The window comparator embedded in the circuit has a high threshold voltage (${V}_{{{\rm{thH}}}}$) of 2.13 V and a low threshold voltage (${V}_{{{\rm{thL}}}}$) of 1.29 V. When the storage capacitor voltage (${V}_{{{\rm{storage}}}}$) exceeds 2.13 V, the internal control circuit engages SW3 while simultaneously disengaging SW2, thereby ensuring that power is drawn exclusively from the storage capacitor to supply the EUU. As the storage capacitor voltage decreases to an intermediate value within the high-low threshold range, SW2 is reactivated, allowing both the EHU and storage capacitor to supply power to the EUU concurrently. SW3 remains engaged until the storage capacitor voltage falls below 1.29 V, at which point it is deactivated, effectively disconnecting the EUU from the power supply. The total energy delivered by the EMU to the EUU can be approximated as follows, yielding a value of approximately 67.88 μJ:

2 $$E=\frac{1}{2}{C}_{{{\rm{sto}}}}\left({V}_{{{\rm{thH}}}}^{2}-{V}_{{{\rm{thL}}}}^{2}\right)$$

By measuring the capacitor voltage under varying input power conditions, it was determined that a 47 μF capacitor can be charged to 2.13 V within approximately 28.9 seconds, completing a full cold start of the entire circuit. Based on the following Eq. 2, the minimum average input power requirement ${P}_{{average}}$ is calculated to be 3.71 μW. Taking into account potential power losses at the EHU and EMU interfaces, the estimated minimum average input power required is approximately 4 μW.

3 $${P}_{{average}}=\frac{{C}_{{{\rm{sto}}}}\left({V}_{{{\rm{thH}}}}^{2}\right)}{2\times T}$$

In the EUU, the control circuit acquires temperature data from an onboard sensor. During the continuous operation of the EUU, it cyclically executes tasks such as temperature data acquisition and transmission via the Bluetooth protocol. The execution interval is configurable, with a default setting of 100 ms, and persists until the EUU is powered down. The collected data is broadcast to a mobile device. The mobile device synchronizes the temperature data with local positioning information, uploads it to the cloud, and displays it on an app. This process provides users with real-time temperature readings and location-based insights.

This mechanism ensures precise control of the energy delivered to the EUU during each cycle. Notably, the EMU does not incorporate conventional switching circuits. This design choice not only reduces the overall circuit board footprint but also enhances the integration of Bluetooth and other RF functionalities within compact devices.

### Reporting summary

Further information on research design is available in the Nature Portfolio Reporting Summary linked to this article.

## Supplementary information

Supplementary Information

Reporting Summary

Transparent Peer Review file

## Data Availability

The data supporting the findings of this study are available within the main text and the Supplementary Information. Additional data are available from the corresponding authors upon request.

## References

[1] Müller, D. et al. Indoor photovoltaics for the Internet-of-Things – a comparison of state-of-the-art devices from different photovoltaic technologies. *ACS Appl. Energy Mater.* **6**, 10404–10414 (2023).

[2] Lübke, D., Hartnagel, P., Hülsbeck, M. & Kirchartz, T. Understanding the thickness and light-intensity dependent performance of green-solvent processed organic solar cells. *ACS Mater. Au* **3**, 215–230 (2023). 38089130 10.1021/acsmaterialsau.2c00070 PMC10176617

[3] Grandhi, G. K. et al. Promises and challenges of indoor photovoltaics. *Nat. Rev. Clean. Technol.* **1**, 132–147 (2025).

[4] Pecunia, V., Occhipinti, L. G. & Hoye, R. L. Z. Emerging indoor photovoltaic technologies for sustainable Internet of Things. *Adv. Energy Mater.* **11**, 2100698 (2021).

[5] Wang, Z. et al. Mechanically robust and stretchable organic solar cells plasticized by small-molecule acceptors. *Science* **387**, 381–387 (2025). 39847644 10.1126/science.adp9709

[6] Zhang, R. et al. Equally high efficiencies of organic solar cells processed from different solvents reveal key factors for morphology control. *Nat. Energy* **10**, 124–134 (2025).

[7] Zhou, X. et al. Over 31% efficient indoor organic photovoltaics enabled by simultaneously reduced trap-assisted recombination and non-radiative recombination voltage loss. *Mater. Horiz.* **10**, 566–575 (2023). 36458496 10.1039/d2mh01229d

[8] Proctor, C. M. & Nguyen, T.-Q. Effect of leakage current and shunt resistance on the light intensity dependence of organic solar cells. *Appl. Phys. Lett*. **106**10.1063/1.4913589 (2015).

[9] Kumar, R. & Gupta, R. Shunts in crystalline silicon PV modules: A comprehensive review of investigation, characterization, and mitigation. *Sol. Energy Mater. Sol. Cells* **277**, 113121 (2024).

[10] Burwell, G. et al. Scaling considerations for organic photovoltaics for indoor applications. *Sol. RRL* **6**, 2200315 (2022).

[11] Cortese, A. J. et al. Microscopic sensors using optical wireless integrated circuits. *Proc. Natl. Acad. Sci.* **117**, 9173–9179 (2020). 32303653 10.1073/pnas.1919677117 PMC7196798

[12] Jahandar, M., Kim, S., Kim, Y. H. & Lim, D. C. Large-area wide bandgap indoor organic photovoltaics for self-sustainable IoT applications. *Adv. Energy Sustain. Res.* **4**, 2200117 (2023).

[13] Dongaonkar, S. et al. Universality of non-Ohmic shunt leakage in thin-film solar cells. *J. Appl. Phys*. **108**. 10.1063/1.3518509.

[14] Larsen-Olsen, T. T., Søndergaard, R. R., Norrman, K., Jørgensen, M. & Krebs, F. C. All printed transparent electrodes through an electrical switching mechanism: A convincing alternative to indium-tin-oxide, silver and vacuum. *Energy Environ. Sci.* **5**, 9467–9471 (2012).

[15] Ma, L.-K. et al. High-Efficiency Indoor Organic Photovoltaics with a Band-Aligned Interlayer. *Joule* **4**, 1486–1500 (2020).

[16] Phuong, L. Q. et al. Quantifying quasi-fermi level splitting and open-circuit voltage losses in highly efficient nonfullerene organic solar cells. *Sol. RRL* **5**, 2000649 (2021).

[17] Wang, J. et al. Intrinsically stretchable organic photovoltaics by redistributing strain to PEDOT:PSS with enhanced stretchability and interfacial adhesion. *Nat. Commun.* **15**, 4902 (2024). 38851770 10.1038/s41467-024-49352-4 PMC11162488

[18] Jayaraman, E. et al. Flexible ITO-free organic solar modules using fully roll-to-roll processable top illumination design. *Adv. Energy Mater*, e04465 10.1002/aenm.202504465.

[19] Zhou, X. et al. Different morphology dependence for efficient indoor organic photovoltaics: the role of the leakage current and recombination losses. *ACS Appl. Mater. Interfaces* **13**, 44604–44614 (2021). 34499484 10.1021/acsami.1c09600

[20] Go, E. et al. Unraveling the origin of dark current in organic bulk heterojunction photodiodes for achieving high near-infrared detectivity. *ACS Photonics* **9**, 2056–2065 (2022).

[21] Park, S. et al. High-performance and stable nonfullerene acceptor-based organic solar cells for indoor to outdoor light. *ACS Energy Lett.* **5**, 170–179 (2020).

[22] Zhang, T. et al. A medium-bandgap nonfullerene acceptor enabling organic photovoltaic cells with 30% efficiency under indoor artificial light. *Adv. Mater.* **34**, 2207009 (2022). 10.1002/adma.202207009 36070897

[23] Li, M., Li, Z., Wang, M., Tang, Z. & Ma, Z. Suppressing shunt and trap-assisted recombination in organic photovoltaic devices for improved indoor light harvesting efficiency. *ACS Appl. Energy Mater.* **7**, 11900–11909 (2024).

[24] Snaith, H. J., Arias, A. C., Morteani, A. C., Silva, C. & Friend, R. H. Charge generation kinetics and transport mechanisms in blended polyfluorene photovoltaic devices. *Nano Lett.* **2**, 1353–1357 (2002).

[25] Li, W. et al. Sparkling hot spots in perovskite solar cells under reverse bias. *ChemPhysMater* **1**, 71–76 (2022).

[26] Simon, M. & Meyer, E. L. Detection and analysis of hot-spot formation in solar cells. *Sol. Energy Mater. Sol. Cells* **94**, 106–113 (2010).

[27] Najafi, L. et al. Reverse-bias and temperature behaviors of perovskite solar cells at extended voltage range. *ACS Appl. Energy Mater.* **5**, 1378–1384 (2022). 35252771 10.1021/acsaem.1c03206 PMC8889533

[28] Jiang, F. et al. Improved reverse bias stability in p–i–n perovskite solar cells with optimized hole transport materials and less reactive electrodes. *Nat. Energy* **9**, 1275–1284 (2024).

[29] Tang, Y. et al. Origin of open-circuit voltage turnover in organic solar cells at low temperature. *Sol. RRL* **4**, 2000375 (2020).

[30] Chiu, F.-C. A review on conduction mechanisms in dielectric films. *Adv. Mater. Sci. Eng.* **2014**, 578168 (2014).

[31] Nau, S., Sax, S. & List-Kratochvil, E. J. W. Unravelling the nature of unipolar resistance switching in organic devices by utilizing the photovoltaic effect. *Adv. Mater.* **26**, 2508–2513 (2014). 24458809 10.1002/adma.201305369

[32] Brus, V. V. On impedance spectroscopy analysis of nonideal heterojunctions. *Semicond. Sci. Technol.* **27**, 035024 (2012).

[33] Keivanidis, P. E., Ho, P. K. H., Friend, R. H. & Greenham, N. C. The dependence of device dark current on the active-layer morphology of solution-processed organic photodetectors. *Adv. Funct. Mater.* **20**, 3895–3903 (2010).

[34] Fu, Y. et al. Enhancing inter-domain connectivity by reducing fractal dimensions: the key to passivating deep traps in organic photovoltaics. *Energy Environ. Sci*. 10.1039/D4EE02961E.

[35] Fernando, P. S. & Mativetsky, J. M. Unambiguous measurement of local hole current in organic semiconductors using conductive atomic force microscopy. * J. Phys. Chem. C.* **127**, 9903–9910 (2023).

[36] Sentein, C., Fiorini, C., Lorin, A. & Nunzi, J.-M. Molecular rectification in oriented polymer structures. *Adv. Mater.* **9**, 809–811 (1997).

[37] Sentein, C. et al. Poling induced improvement of organic polymer device efficiency. *Synth. Met.* **102**, 989–990 (1999).

[38] Padinger, F., Rittberger, R. S. & Sariciftci, N. S. Effects of postproduction treatment on plastic solar cells. *Adv. Funct. Mater.* **13**, 85–88 (2003).

[39] Li, Y. et al. Thermal treatment under reverse bias: Effective tool for polymer/fullerene bulk heterojunction solar cells. *Synth. Met.* **158**, 190–193 (2008).

[40] Devi, B. P., Thiyagu, S. & Pei, Z. Electrical annealing” effect in bulk heterojunction polymer solar cells. *Thin Solid Films* **529**, 54–57 (2013).

[41] Méndez, M., Fernández, D., Viterisi, A., Martínez-Ferrero, E. & Palomares, E. Joule-heating annealing to increase organic solar cells performance: a comparative study. *Appl. Sci.* **12**, 2552 (2022).

[42] Fu, Y. et al. Molecular orientation-dependent energetic shifts in solution-processed non-fullerene acceptors and their impact on organic photovoltaic performance. *Nat. Commun.* **14**, 1870 (2023). 37015916 10.1038/s41467-023-37234-0 PMC10073232

[43] Liao, X. et al. Regulating crystallinity mismatch between donor and acceptor to improve exciton/charge transport in efficient organic solar cells. *Angew. Chem. Int. Ed.* **63**, e202318595 (2024). 10.1002/anie.202318595 38224211

[44] Wu, Q. et al. High-performance organic photovoltaic modules using eco-friendly solvents for various indoor application scenarios. *Joule* **6**, 2138–2151 (2022).

