# Supplementary material for: Reverse-bias enabled mesoscale shunt passivation for organic photovoltaic modules to power miniaturised Ambient IoTs under low-light conditions
Source: Nat Commun. 2026 May 6;17:6109. doi: 10.1038/s41467-026-72623-1 (PMC13357597; doi:10.1038/s41467-026-72623-1)
Supplement: Supplementary file 1 — Supplementary Information [file 41467_2026_72623_MOESM1_ESM.pdf]

Reverse-bias enabled mesoscale shunt passivation for organic photovoltaic modules to power miniaturised Ambient IoTs under low-light conditions

Luhang Xu[1,*], Yuang Fu[1,*], Mianxin Xiao[2], Ho Ming Ng[3], Wenzhi Ma[4], Jun Yan[4], He Yan[3], Xin Li[5,2✉], Wei-Hsin Liao[2,6✉], Xinhui Lu[1✉]

[1] Department of Physics, The Chinese University of Hong Kong, Shatin, Hong Kong SAR 999077, China.

[2] Department of Mechanical and Automation Engineering, The Chinese University of Hong Kong, Shatin, Hong Kong SAR 999077, China.

[3] Department of Chemistry and Hong Kong Branch of Chinese National Engineering Research Centre for Tissue Restoration and Reconstruction, The Hong Kong University of Science and Technology, Clear Water Bay, Kowloon, Hong Kong SAR 999077, China.

[4] Guangdong Basic Research Centre of Excellence for Aggregate Science, School of Science and Engineering, The Chinese University of Hong Kong (Shenzhen), Shenzhen, Guangdong, 518172, China

[5] School of Civil Engineering, Harbin Institute of Technology, Harbin, China.

[6] Institute of Intelligent Design and Manufacturing, The Chinese University of Hong Kong, Shatin, Hong Kong SAR 999077, China.

[*] L. Xu and Y. Fu contributed equally to this work.

✉ email: lixin01@hit.edu.cn; whliao@cuhk.edu.hk; xinhui.lu@cuhk.edu.hk

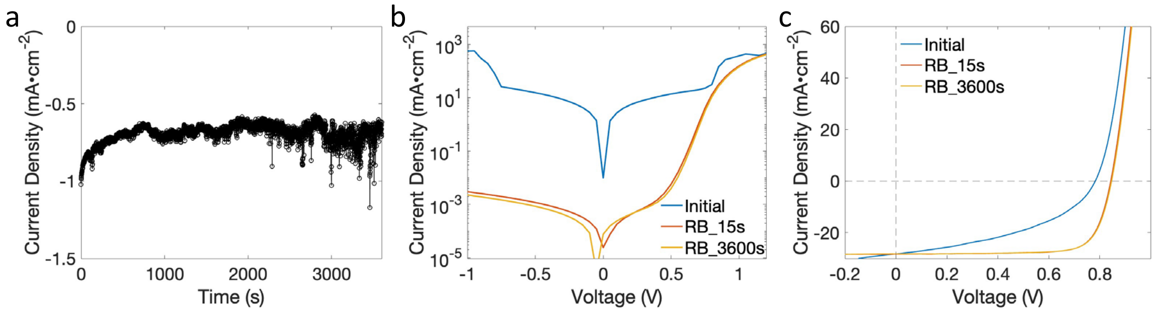

Figure S1 (a) The real-time dark current density extracted during the RB treatment of a PM6:Y6 device for 1 hour. (b) Dark *J*-*V* curves and (c) outdoor light *J*-*V* curves of the same device before (blue lines) and after the RB treatment for 15 s (orange lines) and 1 hour (yellow lines).

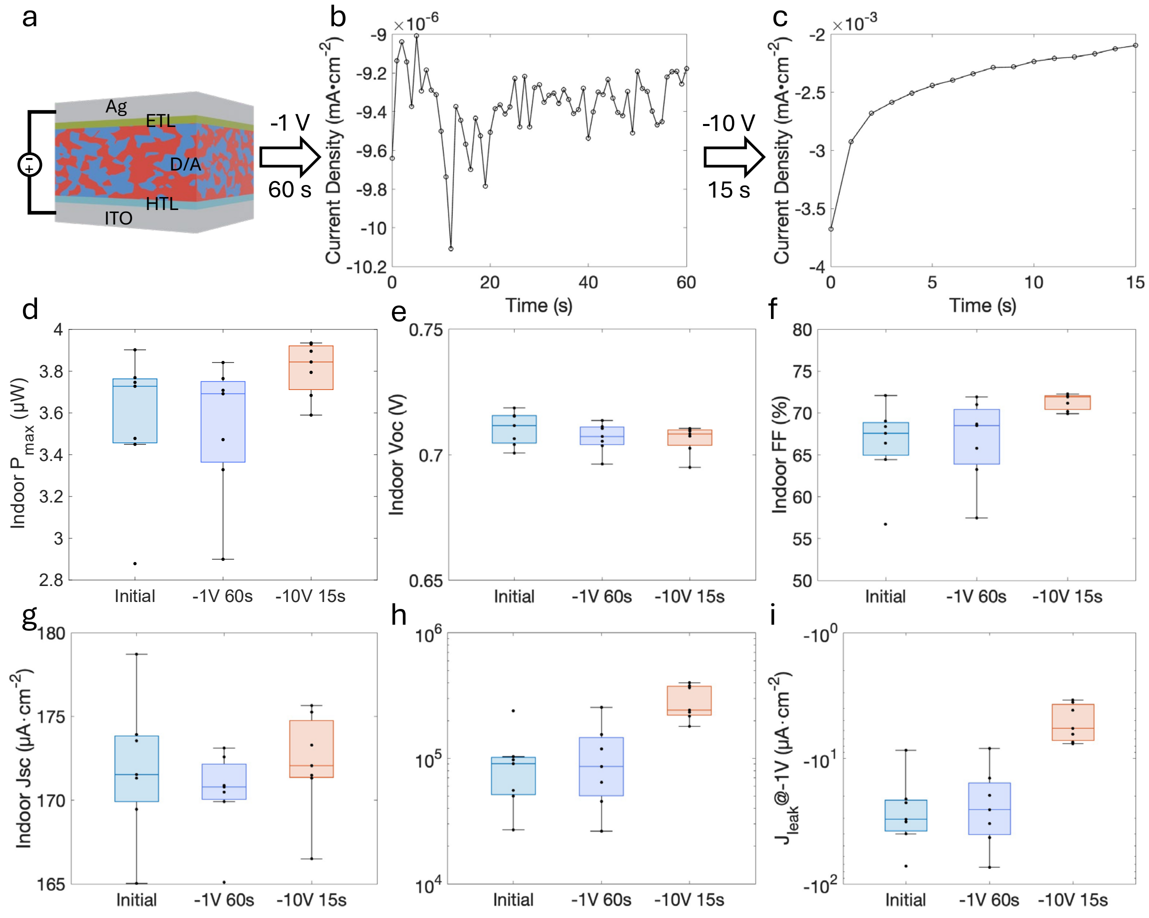

Figure S2 (a) The setup of the RB treatment. The real-time dark current density extracted during the RB treatment under (b) -1 V for 60 s and then (c) -10 V for 15 s. Statistical data of devices from the same batch: (d) P_{max}, (e) V_{OC}, (f) FF, (g) J_{SC}, (h) R_{sh}, and (i) J_{leak} under indoor conditions. The as-fabricated device was first treated at -1 V for 60 s and then treated at -10 V for 15 s. RB treatment at -1 V yields a negligible change in device performance, whereas subsequent RB treatment at -10 V leads to a clear improvement.

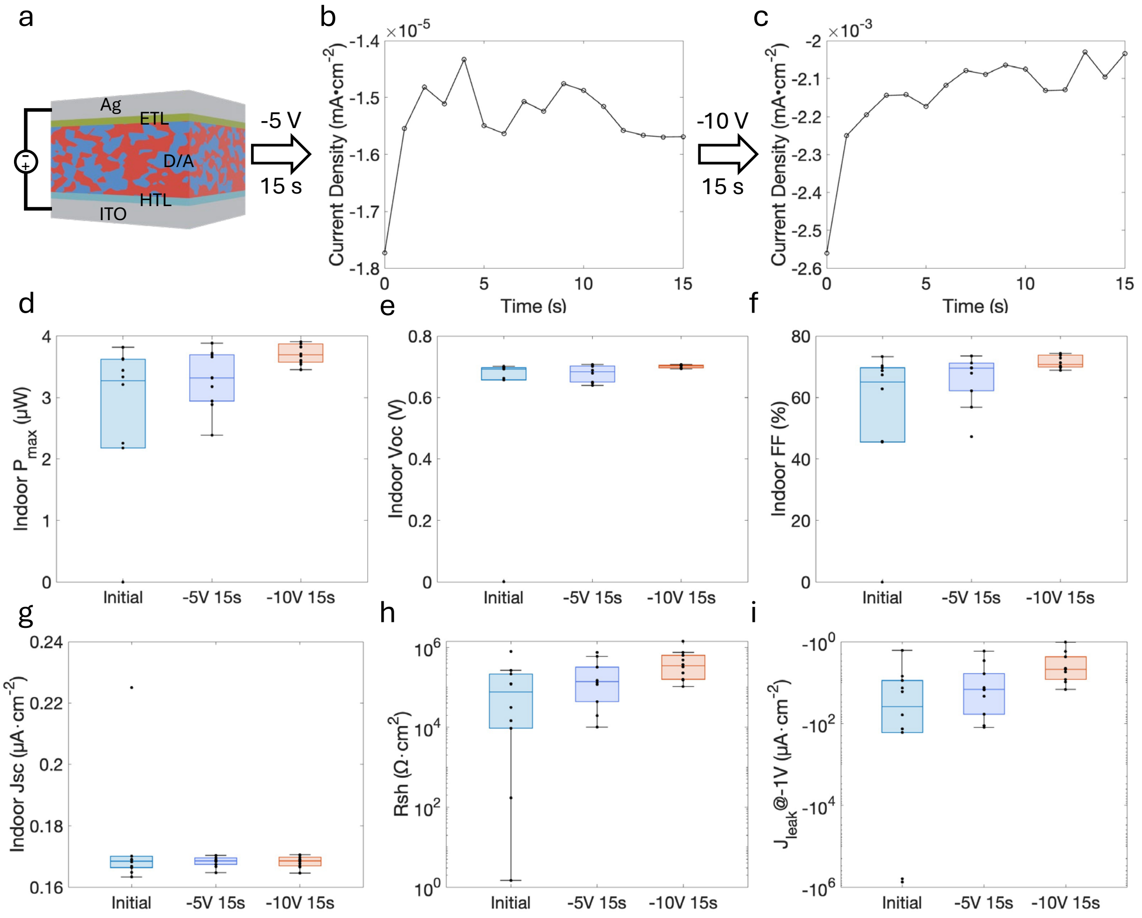

Figure S3 (a) The setup of the RB treatment. The real-time dark current density extracted during the RB treatment under (b) -5 V for 15s and then (c) -10 V for 15s. Statistical data of devices from the same batch: (d) P_{max}, (e) V_{OC}, (f) FF, (g) J_{SC}, (h) R_{sh}, and (i) J_{leak} under indoor conditions. The as-fabricated device was first treated under -5 V for 15 s and then treated under -10 V for 15 s. RB treatment at -5 V partially cures the devices, which can be further enhanced by subsequent treatment at -10 V.

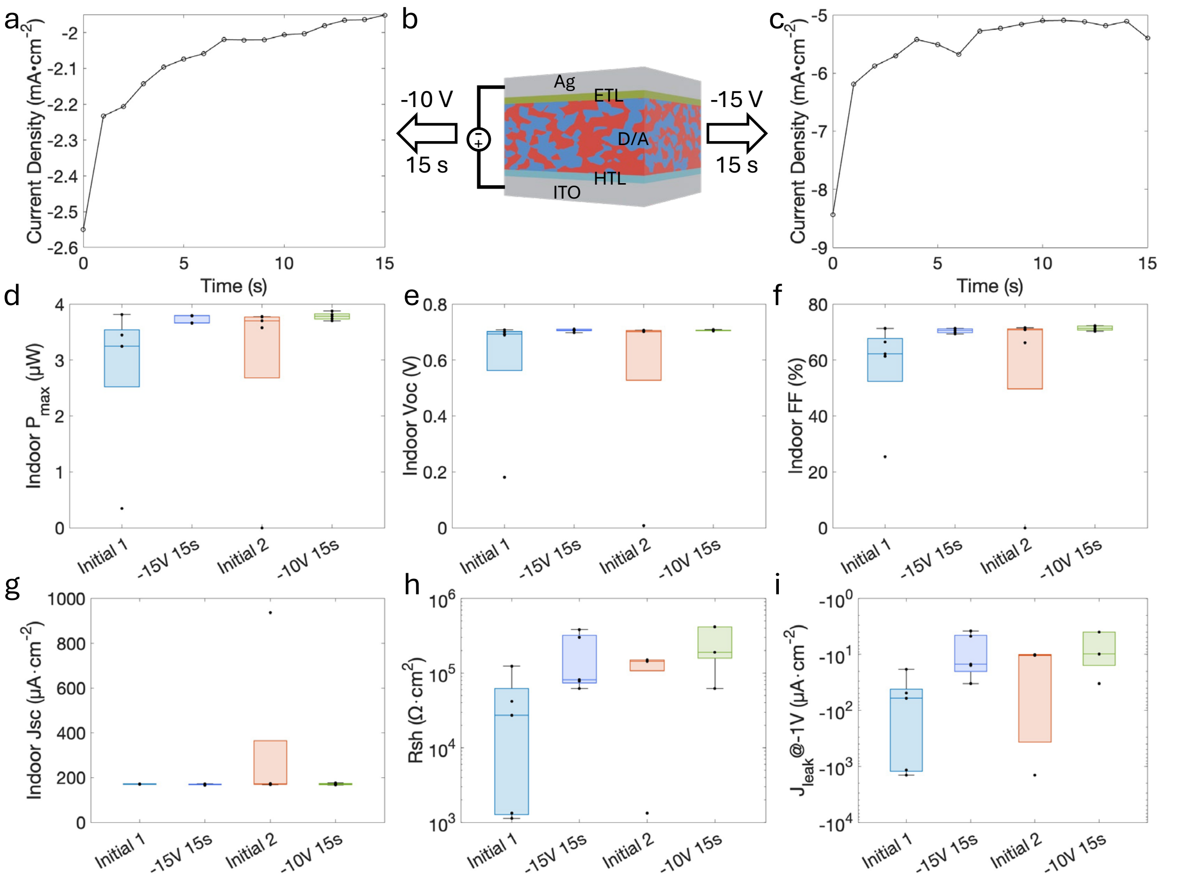

Figure S4 The real-time dark current density extracted during the RB treatment under (a) -15 V for 15s and (c) -10 V for 15s. (b) The setup of the RB treatment. Statistical data of devices from the same batch: (d) P_{max}, (e) V_{OC}, (f) FF, (g) J_{SC}, (h) R_{sh}, and (i) J_{leak} under indoor conditions. The as-fabricated devices (Initial 1) were treated under -15 V for 15 s. The as-fabricated devices (Initial 2) were treated under -10 V for 15 s. RB treatment at -15 V has a similar effect to that at -10 V.

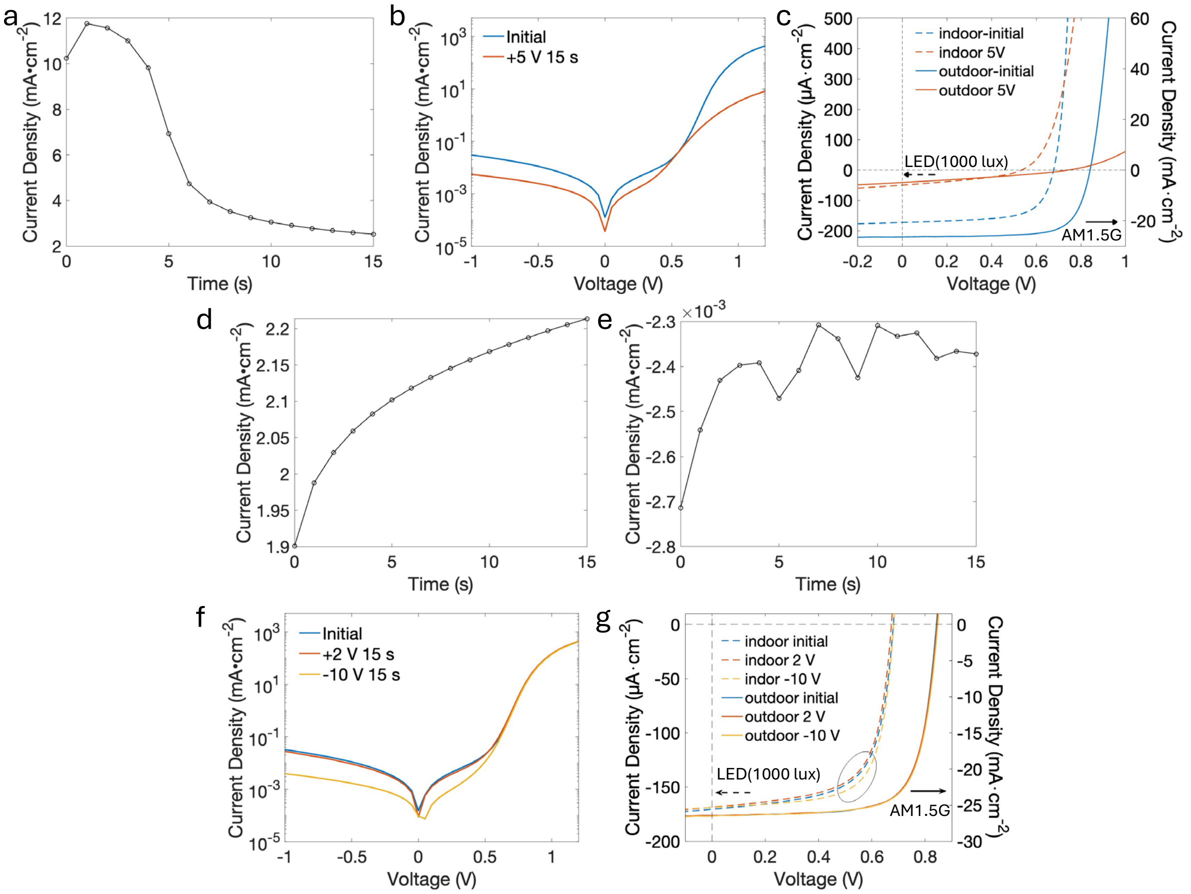

Figure S5 (a) The real-time dark current density extracted during the forward-bias (FB) treatment under +5 V for 15 s. (b) Dark *J-V* curves of the same as-fabricated device before and after FB treatment under +5 V. (c) Indoor (dashed lines) and outdoor (solid lines) light *J-V* curves of the same device before (blue lines) and after the FB treatment (orange lines) under +5 V. (d) The real-time dark current density extracted from the same device during the FB treatment under (d) +2 V for 15 s and then (e) -10 V for 15 s. (b) Dark *J-V* curves of the same as-fabricated device before and after treatment under +2 V and -10 V. (c) Indoor (dashed lines) and outdoor (solid lines) light *J-V* curves of the same device before the treatment (blue lines), after the FB treatment under +2 V (orange lines) and after the subsequent RB treatment under -10 V (gold lines). FB treatment at +2 V has a negligible impact on the device performance, while further increasing the FB to +5 V degrades the device.

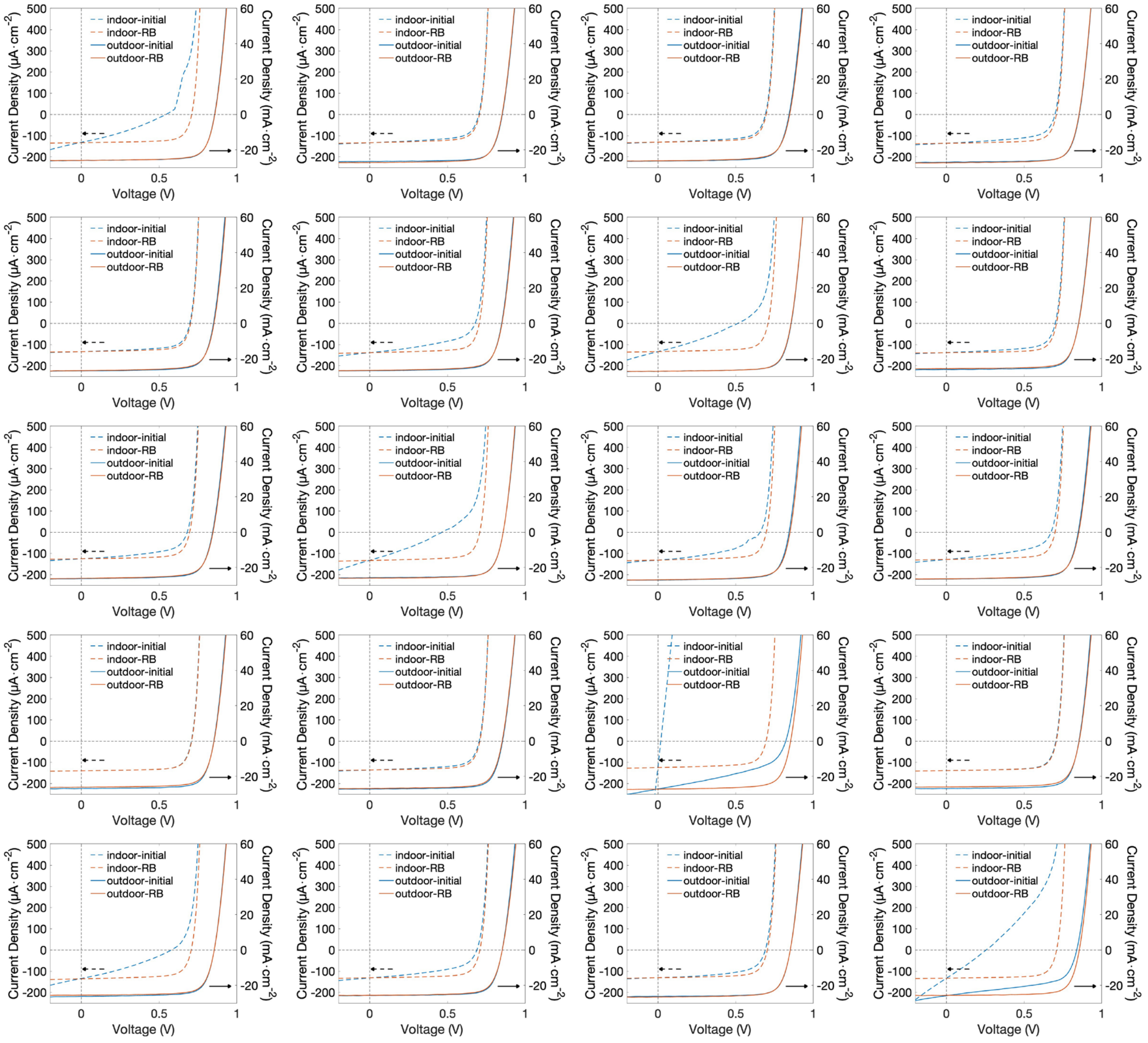

Figure S6 Indoor (dashed lines) and outdoor (solid lines) light *J-V* curves of the same device before (blue lines) and after the RB treatment (orange lines).

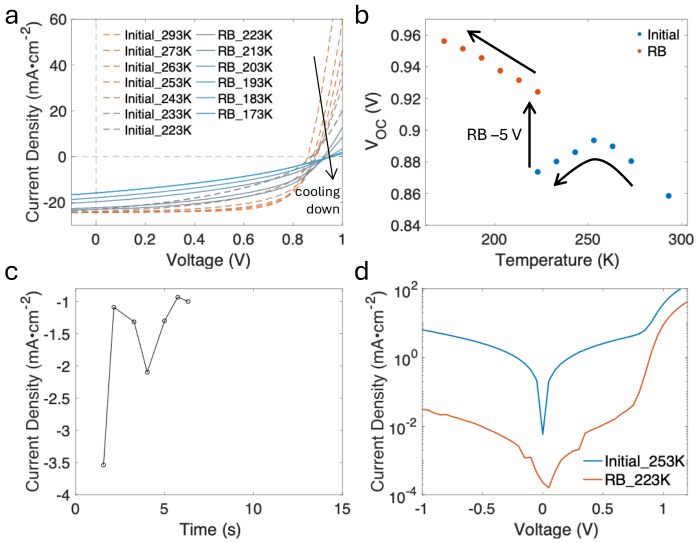

Figure S7 (a) Temperature-dependent light *J-V* curves of the same device before (dashed line) and after (solid line) the RB treatment. (b) V_{OC}-temperature plot of the same device before and after the RB treatment. (c) The real-time dark current density during the RB treatment at 223K. (d) The dark *J-V* curves of the same device before and after the RB treatment. The initial curve was measured at 253K, corresponding to the V_{OC} turnover temperature. The curve after the RB treatment was measured at 223K, the temperature at which the RB treatment was applied. In the temperature-dependent measurement, the device with low shunt resistance exhibits a turnover at 253 K. A reverse bias was subsequently applied at 223 K to eliminate the shunt pathway. Owing to inefficient heat dissipation in the encapsulated device, a relatively lower reverse bias (–5 V) with a shorter duration (5 s) was chosen to avoid device overheating. After the RB treatment, the leakage current is effectively suppressed, resulting in an increased V_{OC} at 223 K and the recovery of a linear relationship between V_{OC} and temperature.

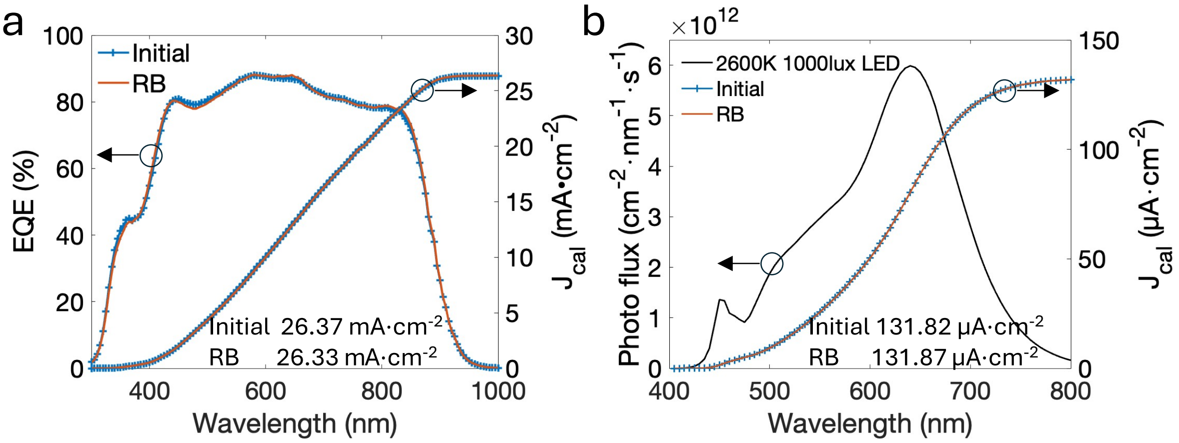

Figure S8 (a) The external quantum efficiency (*EQE*) curves with their corresponding calculated outdoor J_{SC}s. (b) The photon flux spectrum (black line) of the white LED (2600K, 1000lux) and the calculated indoor J_{SC}s.

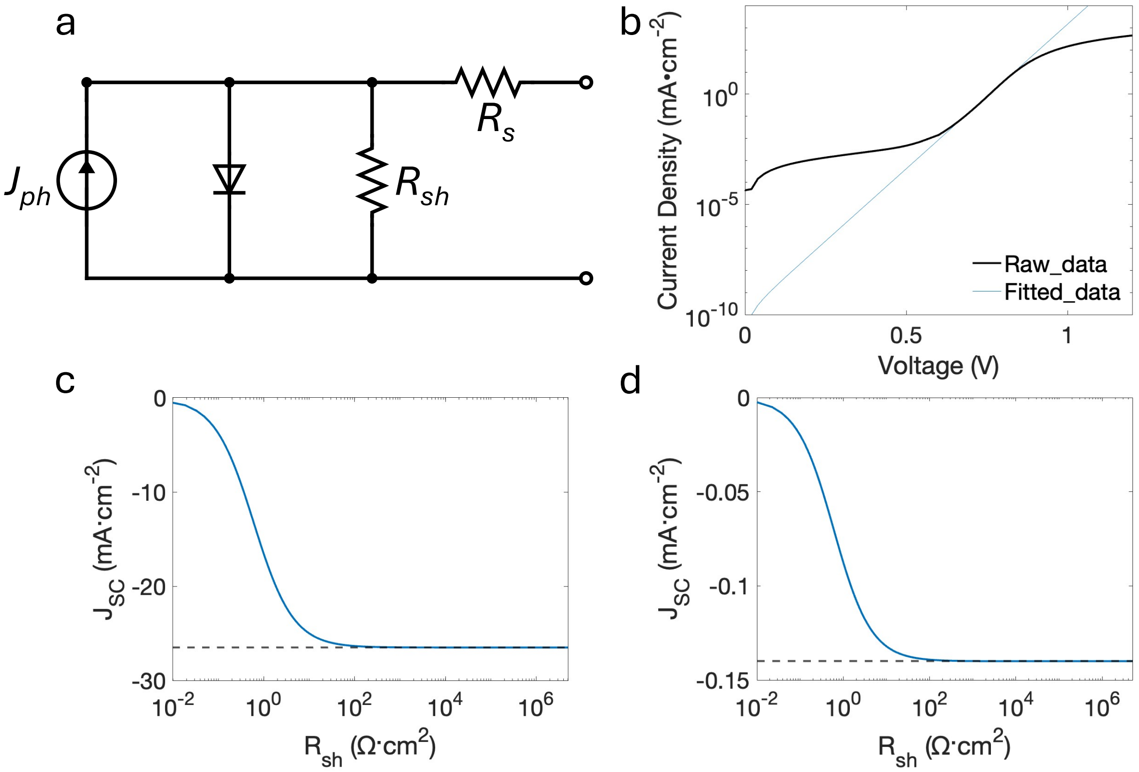

Figure S9 (a) The equivalent circuit model for photovoltaics. The current source (photocurrent), the diode, and the shunt resistance are connected in parallel, while the series resistance is connected in series with the other components. (b) The fitting of the 'RB' dark current in **Figure 1b** within the diode-current dominant (exponential) region. The simulation of J_{SC} (blue line) with different R_{sh} under outdoor (c) and indoor (d) conditions.

To figure out the influence of leakage current on J_{SC}, we simulate J_{SC} with different R_{sh} as follows:

1) The ideal diode equation (**eq1**) is used to fit the exponential current region in the dark current.

$$J = J_0 \left[exp\left(\frac{qV}{nkT}\right) - 1 \right] \qquad \text{(eq1)}$$

The dark saturation current J_0 and ideality factor n can be obtained from the fitting result, which are $1.15 \times 10^{-10}\ mA \cdot cm^{-2}$ and 1.19 for the 'RB' dark current in **Figure 1b**, respectively.

2) With photoexcitation, the current across the device is the sum of the photocurrent, intrinsic dark current and shunt leakage current, as presented in **eq2**:

$$J = J_{ph} - \frac{V+JR_s}{R_{sh}} - J_0 \left\{ exp\left[\frac{q}{nkT}(V + JR_s)\right] - 1 \right\} \qquad \text{(eq2)}$$

In the short circuit condition, **eq 2** to simplify the calculation (**eq 3**).

$$J = J_{ph} - \frac{JR_s}{R_{sh}} - J_0\left[exp\left(\frac{qJR_s}{nkT}\right) - 1\right] \quad \text{eq3}$$

3) For the device in **Figure 1c**, we assume the photocurrents are 26.5 and 0.140 $mA \cdot cm^2$ under outdoor and indoor conditions, respectively. Then, by inserting the device parameters R_s, J_0, and n into **eq 3**, the dependence of J_{SC} on R_{sh} can be simulated, as illustrated in **Figure S9c** and **d**. From the results, it becomes apparent that J_{sc} is independent of R_{sh} when R_{sh} becomes larger than 100 $\Omega \cdot cm$ as indicated by the grey dashed lines in **Figure S9c** and **d**.

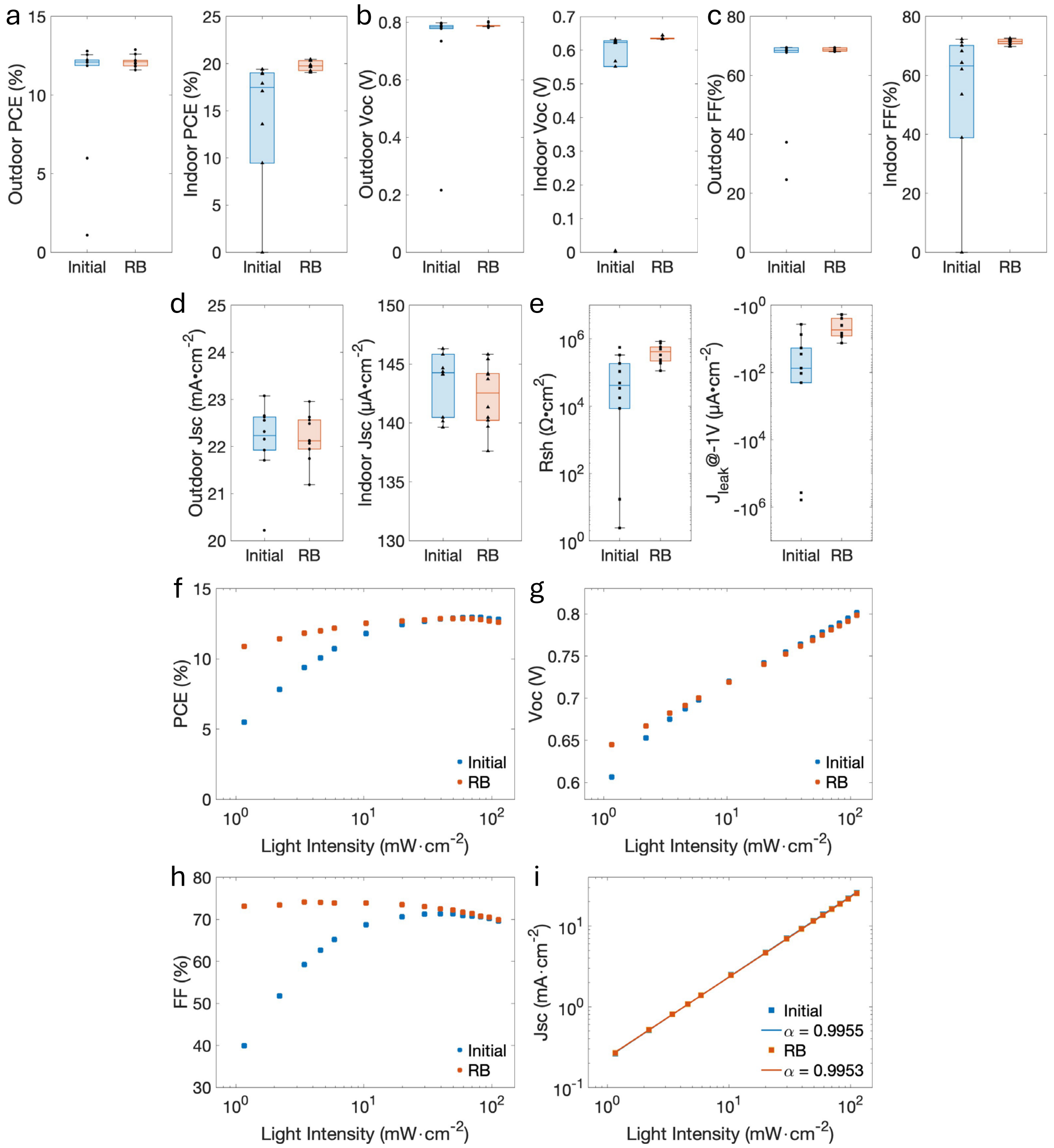

Figure S10 Statistics of PM6:Y6 (CB) device performance: (a) *PCE*, (b) V_{OC}, (c) *FF*, (d) J_{SC}, (e) R_{sh}, and J_{leak}. Light intensity-dependent performance of the same device before and after the RB treatment: (f) *PCE*, (g) V_{OC}, (h) *FF*, and (i) J_{SC}.

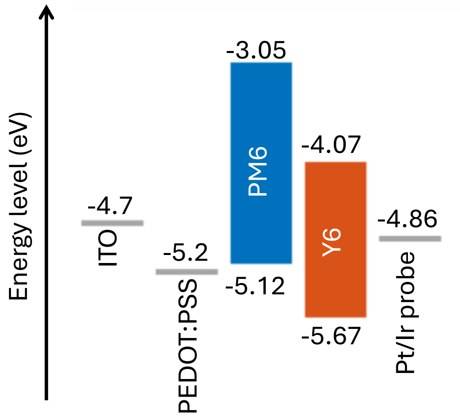

Figure S11 Energy level diagram of ITO/PEDOT:PSS/PM6:Y6/(Pt/Ir) probe used in the c-AFM measurement.[1,2]

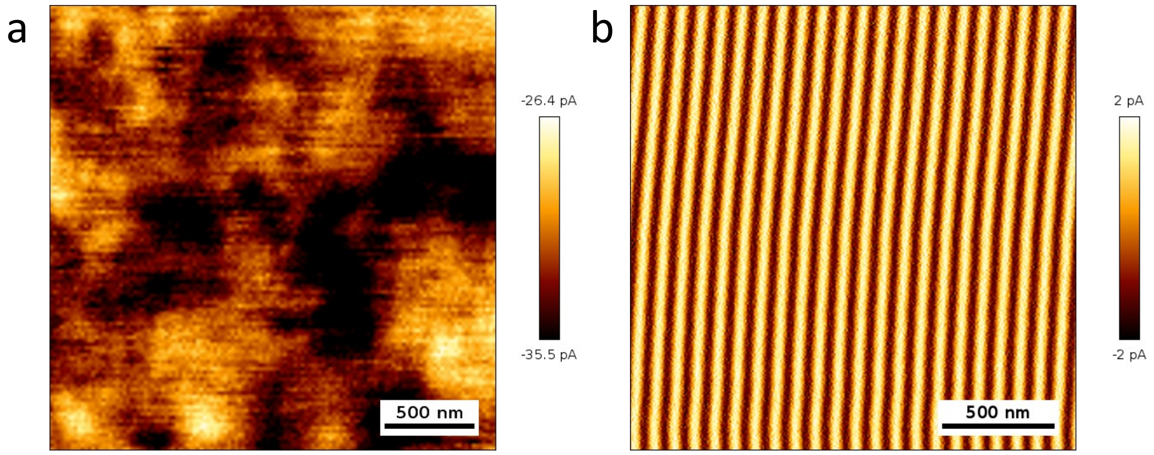

Figure S12 c-AFM mappings of (a) pure PM6 and (b) pure Y6 films deposited on PEDOT:PSS/ITO. Due to energy level alignment, the current falls to almost 0 pA in the pure Y6 film so that c-AFM mapping only shows background noise.

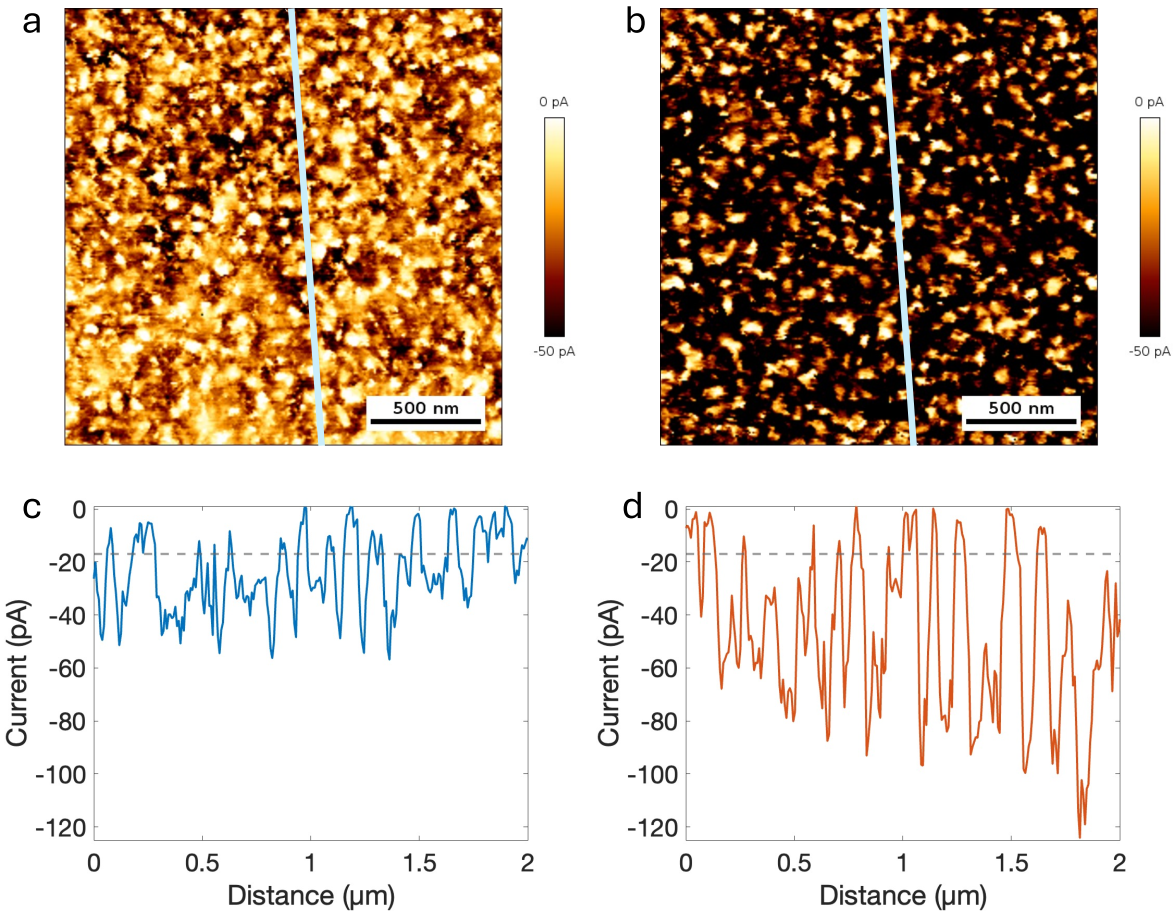

Figure S13 The c-AFM current mappings (a) without and (b) with the RB treatment. The linecut profiles (c) without and (d) with the RB treatment along blue lines in (a) and (b), respectively.

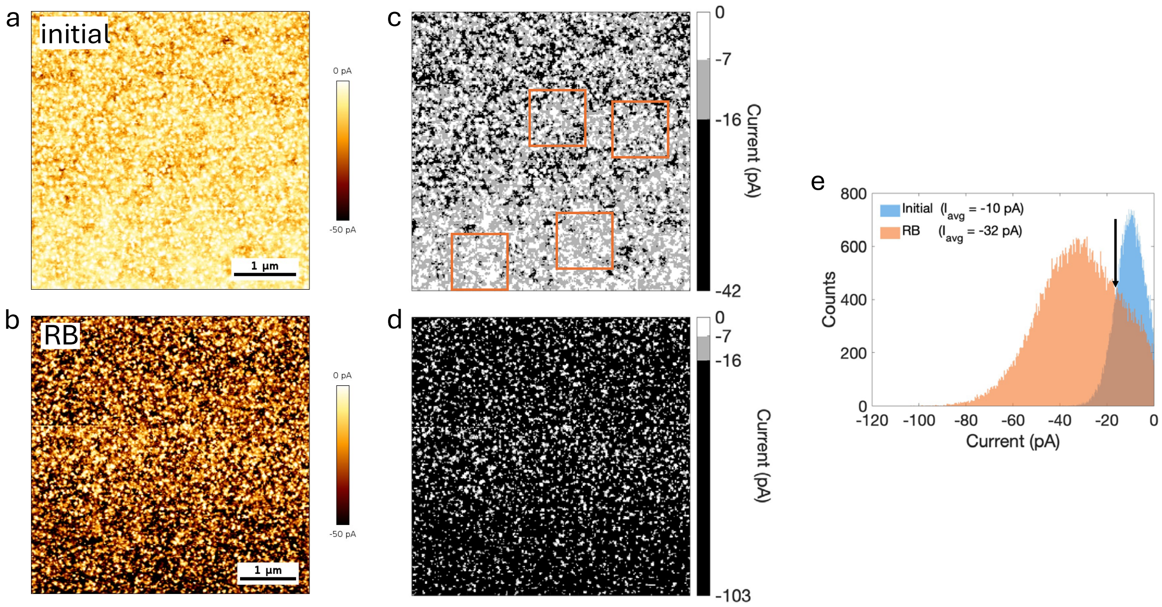

Figure S14 Current mappings (5×5 μm^2) of (a) initial and (b) RB-treated PM6:Y6 (CB) devices. (c) and (d) The re-colored c-AFM mappings corresponding to (a) and (b). The orange box in (c) with a size of 1μm×1μm is used to highlight the presence of mesoscale Y6-rich agglomerates. (e) Current histograms of (a) and (b).

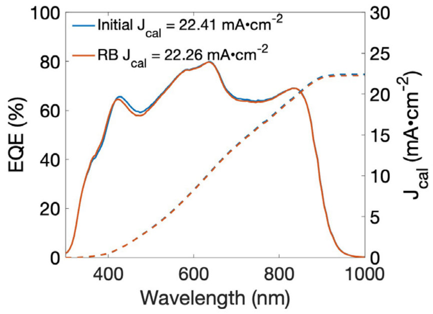

Figure S15 EQE curves with their corresponding calculated outdoor J_{SC}s (dot lines) for the PM6:Y6 (CB) device before and after the RB treatment.

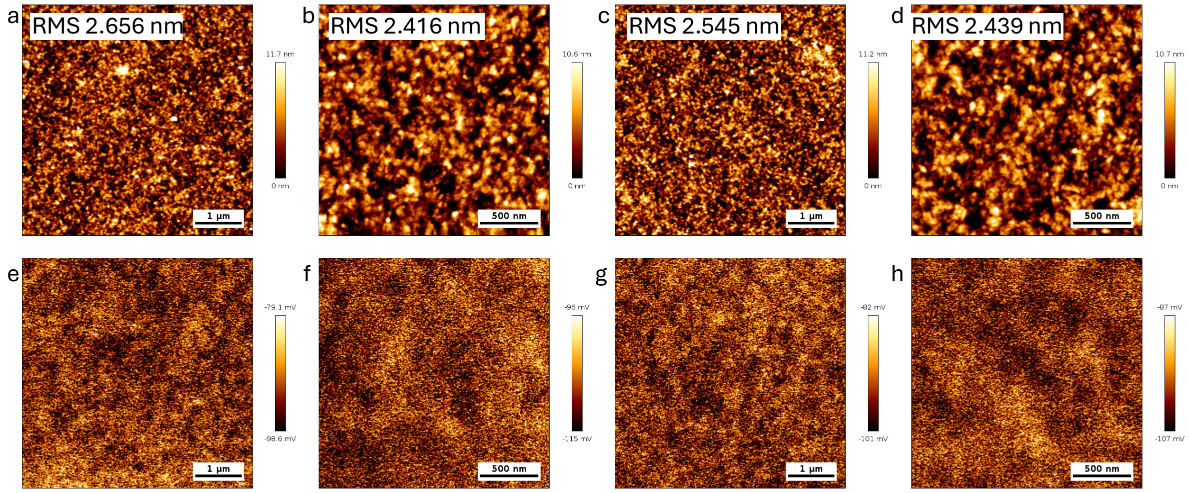

Figure S16 Topography and contact potential difference (CPD) mappings of the initial PM6:Y6 (CB) device with a mapping size of (a,e) 5×5 μm^2 and (b,f) 2×2 μm^2. The corresponding mappings for the RB-treated device with a mapping size of (c,g) 5×5 μm^2 and (d,h) 2×2 μm^2.

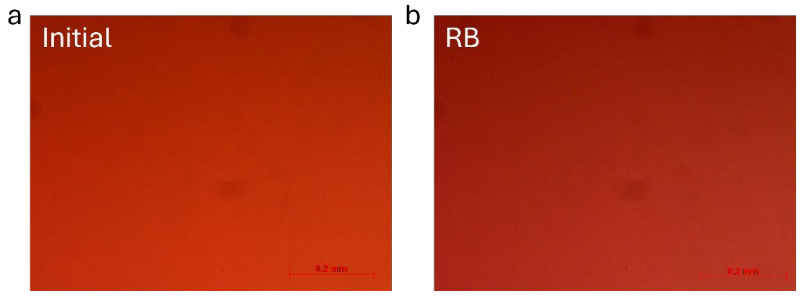

Figure S17 The optical microscopy images of the active layers (PM6:Y6 in CB) (a) without RB treatment and (b) with RB treatment. This measurement was carried out on the initial and RB devices after peeling off the electrode and electron transport layer under a 10× objective lens. The lightly shaded regions originate from dust on the objective lenses as they appear at the same location in every image.

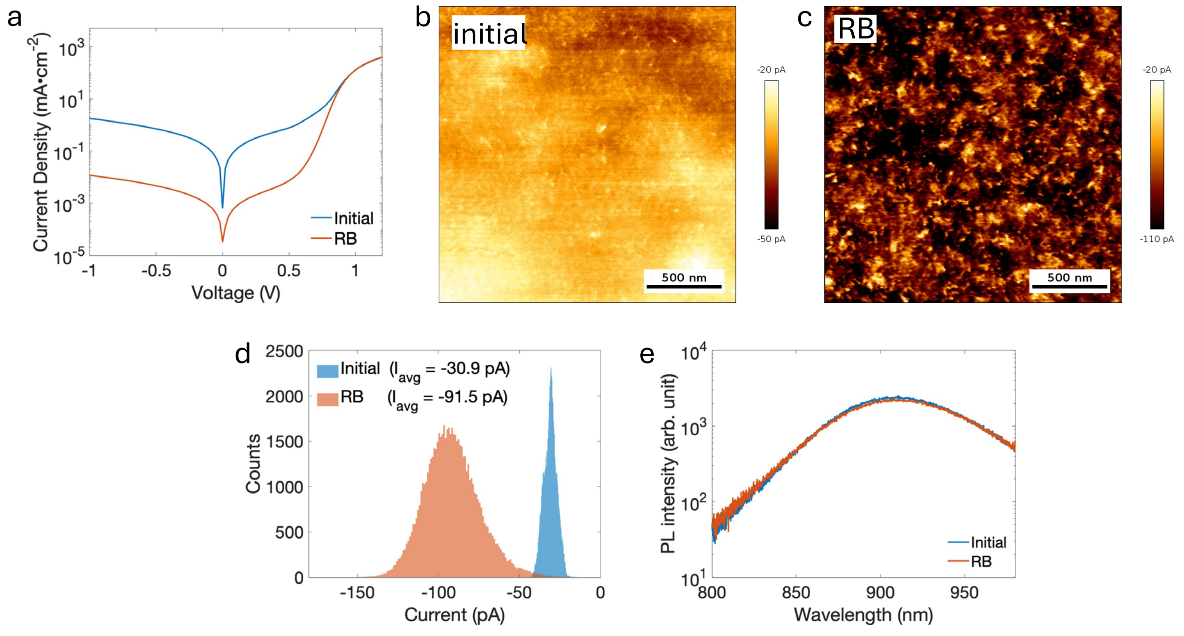

Figure S18 (a) Dark *J*-*V* curves of PM6:Y6 (CF) devices without and with the RB treatment for the c-AFM measurement. Current mappings (2×2 μm^2) of (b) initial and (c) RB-treated devices. (d) Current histograms of the corresponding current mappings in (b) and (c). (e) The corresponding micro-PL spectra.

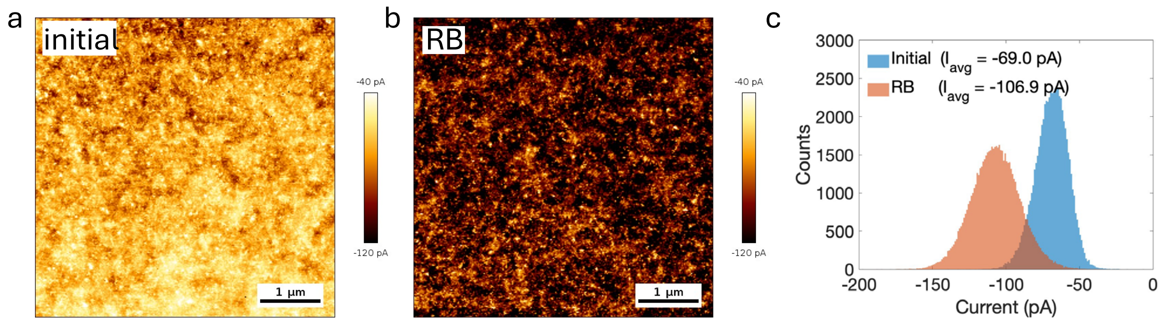

Figure S19 Current mappings (5×5 μm^2) of (a) initial and (b) RB-treated devices. (c) Current histograms of the corresponding current mappings in (a) and (b).

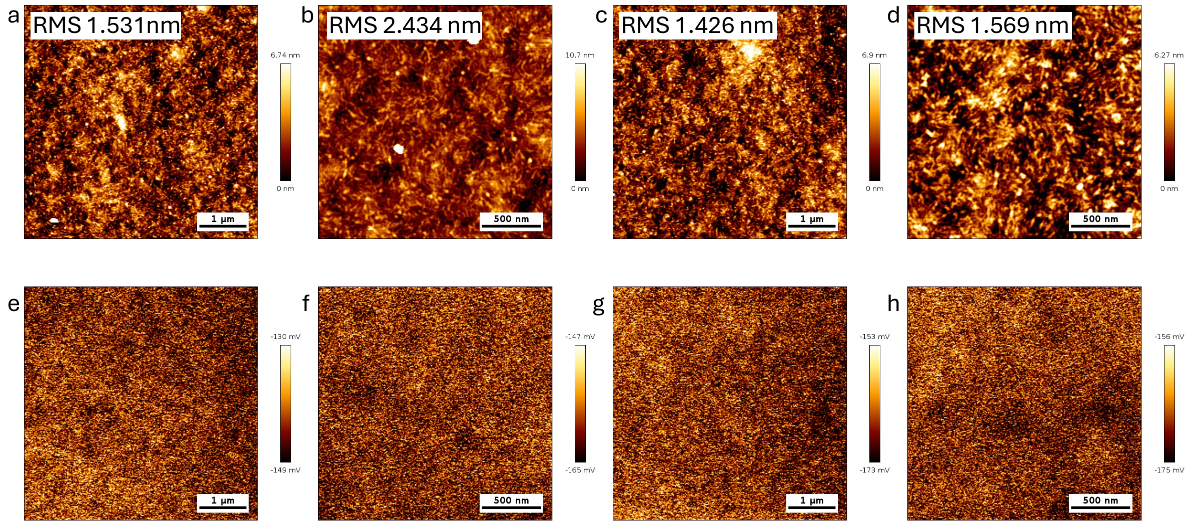

Figure S20 Topography and CPD mappings of the initial PM6:Y6 (CF) device with a mapping size of (a,e) 5×5 μm^2 and (b,f) 2×2 μm^2. The corresponding mappings of the RB-treated device with a mapping size of (c,g) 5×5 μm^2 and (d,h) 2×2 μm^2.

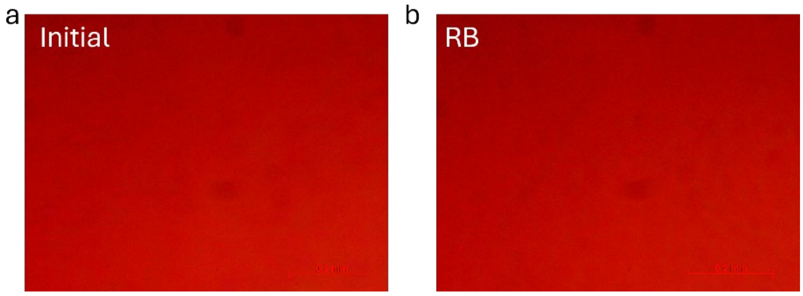

Figure S21 The optical microscopy images of the active layers (PM6:Y6 in CF) (a) without RB treatment and (b) with RB treatment. This measurement was carried out on the initial and RB devices after peeling off the electrode and electron transport layer under a 10× objective lens. The lightly shaded regions originate from dust on the objective lenses as they appear at the same location in every image.

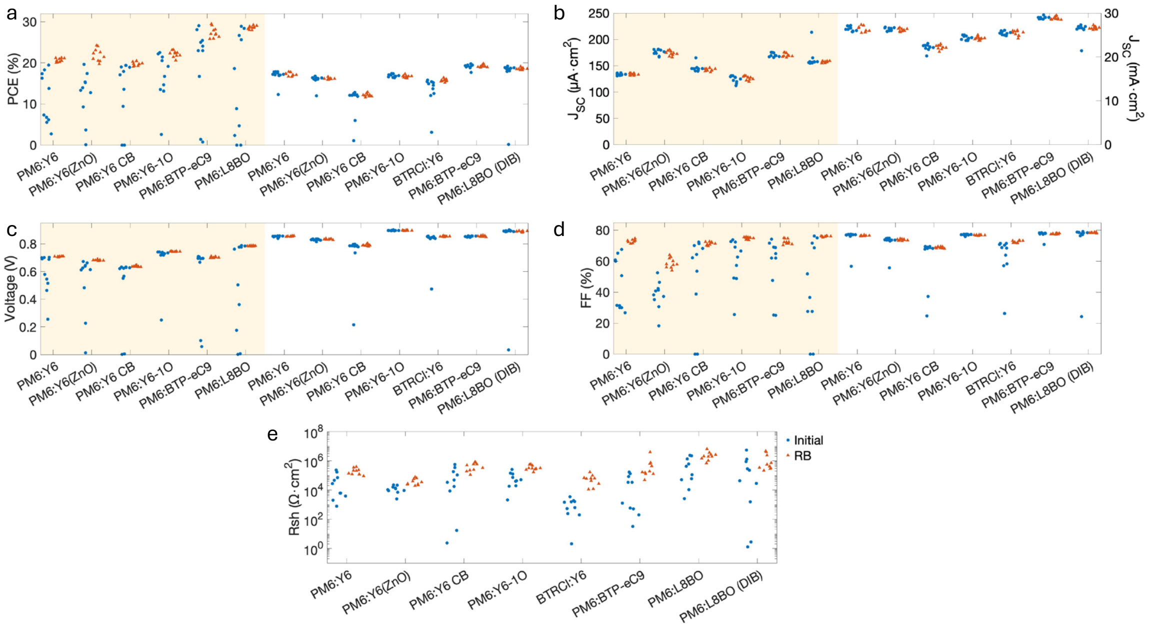

Figure S22 Indoor (shaded) and outdoor (transparent) performance statistics of devices based on different NFAs and CTLs before (blue dots) and after (orange dots) the RB treatment: (a) *PCE*, (b) J_{SC}, (c) V_{OC}, (d) *FF*, and (e) R_{sh}.

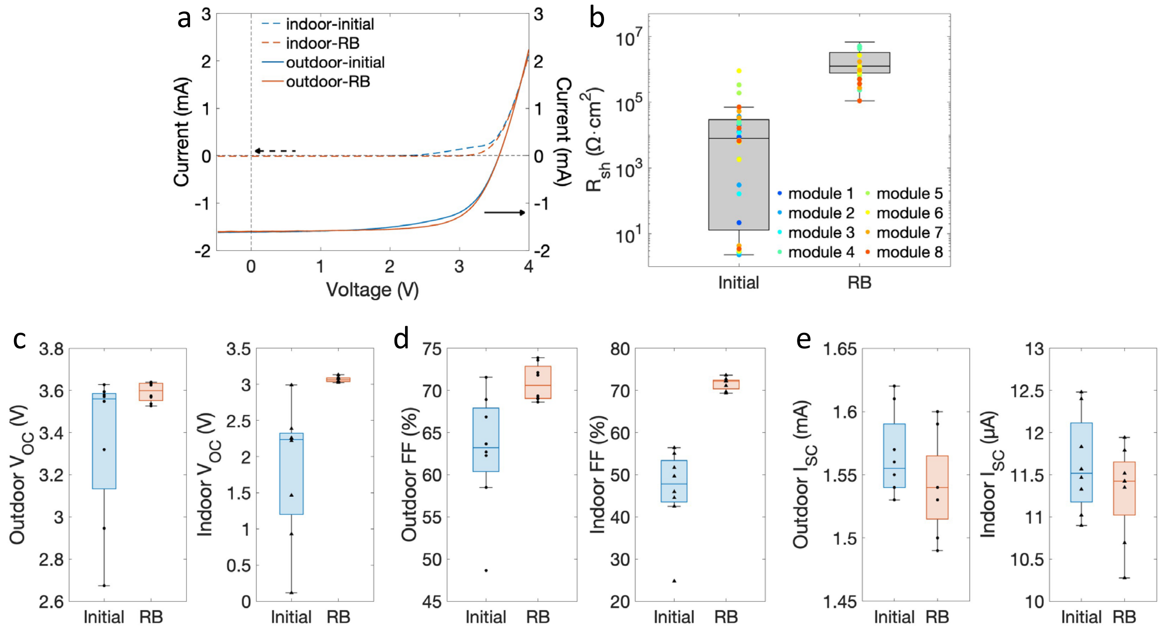

Figure S23 (a) Indoor (dashed lines) and outdoor (solid lines) light *J-V* curves of the same module before (blue lines) and after the RB treatment (orange lines). Statistical data of modules: (b)R_{sh}, (c) V_{OC}, (d) *FF*, and (e) I_{SC}.

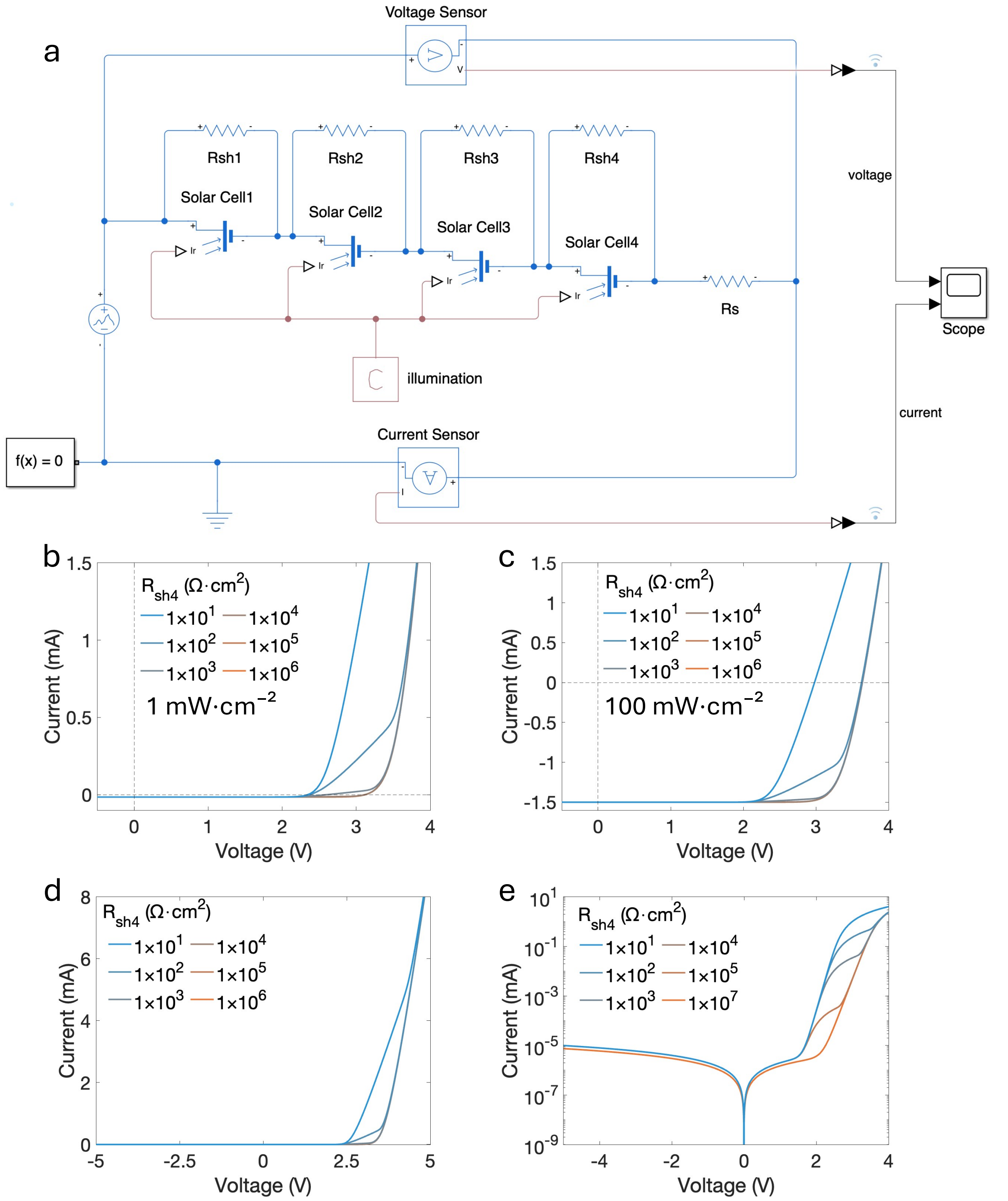

Figure S24 *(*a) Schematics of the four-diode model. The simulated *J-V* curves with different R_{sh4} under illumination of (b) 1 mW·cm^{-2} and (c) 100 mW·cm^{-2}, respectively. Dark *J-V* curves with different R_{sh4} and y-axis plotted in (d) linear scale and (e) log scale, respectively. V_{OC}, I_{SC}, ideality factor, and R_s of the solar cell (diode) are set to 0.91 V, 1.50 mA, 1.1, and 1.9 Ω·cm^2, respectively.

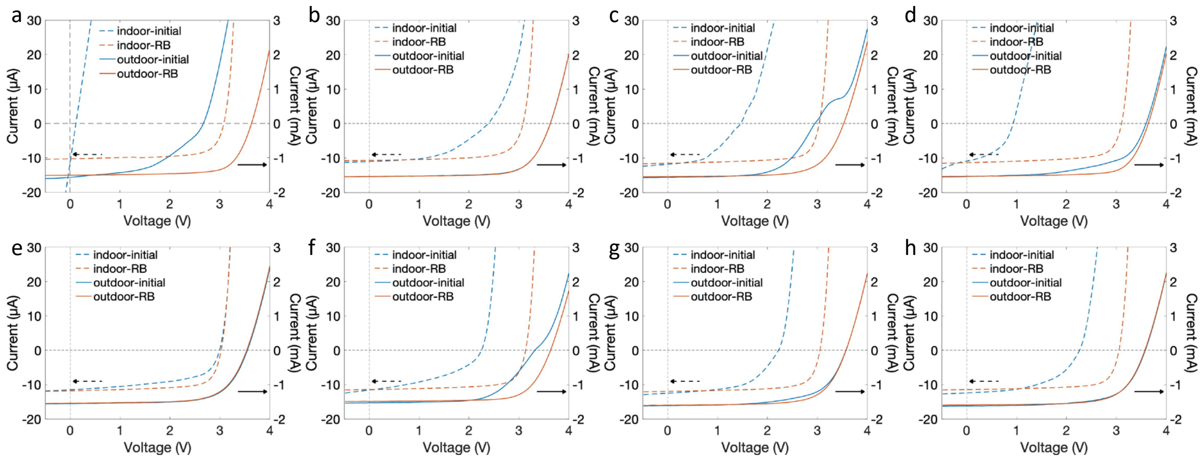

Figure S25 Indoor (dashed lines) and outdoor (solid lines) light *J*-*V* curves of the 8 independent modules before (blue lines) and after RB treatment (orange lines).

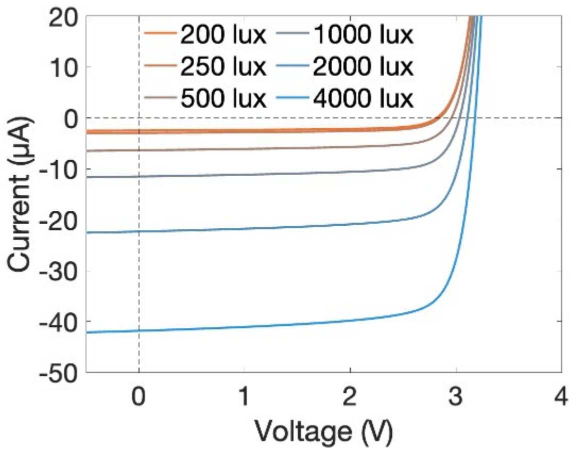

Figure S26 *J*-*V* curves of an RB-treated OPV module under an illuminance range of 4000-200 lux.

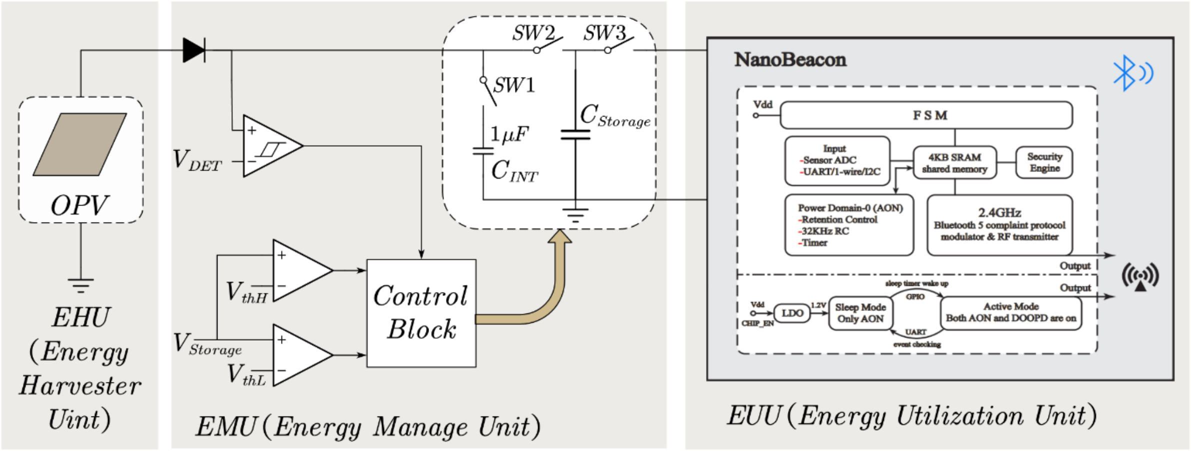

Figure S27 Block Diagram of the A-IoT temperature sensor.

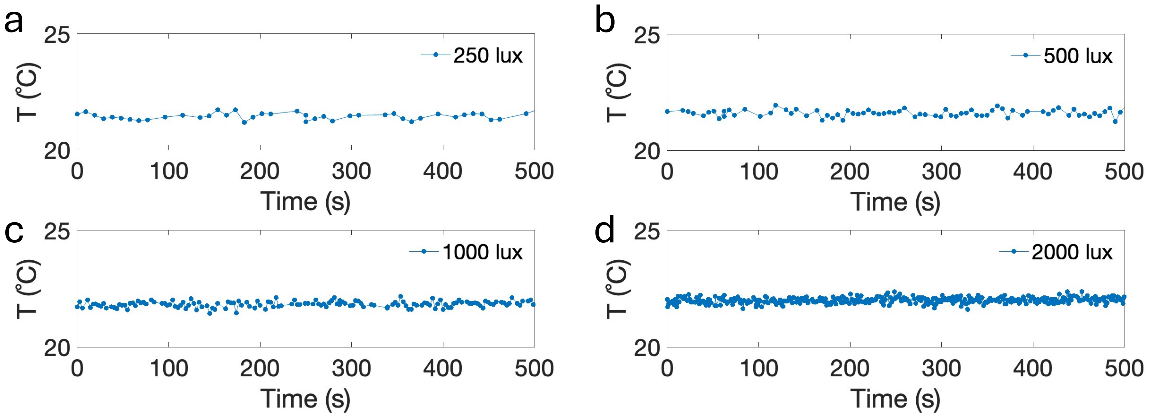

Figure S28 Real-time temperature monitoring under the illuminance of (a) 250 lux, (b) 500 lux, (c) 1000 lux, and (d) 2000 lux.

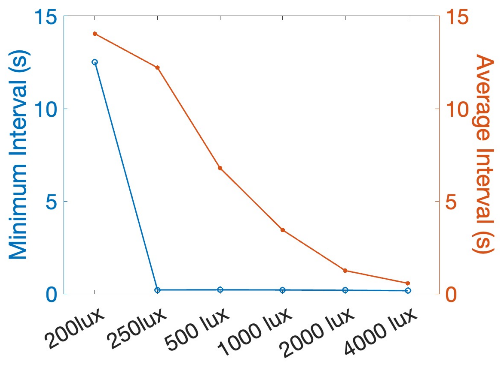

Figure S29 The minimal and average execution interval of the sensor under various illuminances.

Table S1. A summary of device characteristics measured in different conditions

Treat	Light	*PCE* (%)	V_{OC} (V)	J_{SC}[a] (mA·cm^{-2} or µA·cm^{-2})	*FF* (%)	R_{sh}[e] (Ω·cm^2)	J_{leak} (µA/cm^2)
Initial[b]	AM1.5G[d]	17.23	0.848	26.76	76.01	12440	-143.77
RB[b]	AM1.5G[d]	16.95	0.850	26.61	74.96	156351	-8.85
Initial[b]	2600 K 1000 lux[d]	13.01	0.678	138.34	44.97	12440	-143.77
RB[b]	2600 K 1000 lux[d]	21.78	0.704	138.29	72.51	156351	-8.85
Initial[c]	AM1.5G[d]	16.65±1.94	0.849 ±0.008	26.51 ±0.45	73.94 ±8.14	43214 ±57138	-1211.21 ±4006.53
RB[c]	AM1.5G[d]	17.04±0.39	0.852 ±0.003	26.37 ±0.57	75.80 ±0.90	206561 ±92674	-8.58 ±3.41
Initial[c]	2600 K 1000 lux[d]	13.37±6.39	0.602 ±0.174	133.00 ±4.15	51.08 ±16.65	43214 ±57138	-1211.21 ±4006.53
RB[c]	2600 K 1000 lux[d]	20.74±0.86	0.704 ±0.005	133.16 ±4.15	72.94 ±1.27	206561 ±92674	-8.58 ±3.41

[a] The unit of J_{sc} is mA·cm^{-2} and µA·cm^{-2} for outdoor and indoor conditions, respectively.

[b] Detailed parameters of the device in **Figure 1b-c**.

[c] Average values and standard deviations of device parameters in **Figure 1d-h** obtained from 20 independent devices.

[d] Simulator with AM1.5G spectrum and 100mW·cm^{-2} intensity as the outdoor light source; white LED with 2600 K color temperature and 1000 lux illuminance as the indoor light source.

[e] R_{sh} obtained from the inverse slope of dark *J-V* curves at 0V.

Table S2 A summary of device performance for PM6:Y6 devices processed by CB

OPV	Treat	Light	*PCE* (%)	V_{OC} (V)	*FF* (%)	J_{SC} (mA·cm^{-2} or μA·cm^{-2})	R_{sh} (Ω·cm^2)	J_{leak}@-1 (μA/cm^2)
PM6:Y6 in CB[a]	Initial	AM1.5 G	10.51 ±3.66	0.725± 0.171	61.26± 15.41	22.14 ±0.74	129860± 176196	-98935.78 ±205244.82
	RB		12.15 ±0.36	0.789± 0.006	68.82± 0.54	22.18 ±0.48	433155± 237307	-5.8±3.49
	Initial	3000 K 1000 lux	13.47 ±7.36	0.489± 0.244	50.11± 26.81	145.47 ±6.9	129860± 176196	-98935.78 ±205244.82
	RB		19.78 ±0.48	0.636± 0.004	71.44± 0.95	142.27 ±2.62	433155± 237307	-5.8±3.49

[a] Average values and standard deviations of device parameters in **Figure S9a-e** obtained from 10 independent devices.

Table S3 A summary of device performance for devices employing different NFAs and CTLs, in different device structures.

OPV	Label	Light	*PCE* (%)	V_{OC} (V)	*FF* (%)	J_{SC}[a] (mA·cm^{-2} or µA·cm^{-2})	R_{sh}[e] (Ω·cm^2)	J_{leak} (µA/cm^2)
ITO/PEDOT:PSS/PM6:Y6-1O/PDINO/Ag	Initial	AM1.5G	16.86 ±0.33	0.896 ±0.002	77.07 ±0.44	24.36 ±0.47	76900 ±73540	-103.32 ±176.66
	RB		16.68 ±0.28	0.896 ±0.002	76.78 ±0.16	24.2 ±0.37	352226 ±133766	-4.71 ±1.33
	Initial	2600 K 1000 lux	16.64 ±5.77	0.683 ±0.145	59.77 ±14.44	124.09 ±6.12	123537 ±104089	-103.32 ±176.66
	RB		22.15 ±0.75	0.745 ±0.001	74.95 ±0.68	126.23 ±3.58	305589 ±183841	-4.71 ±1.33
ITO/PEDOT:PSS/BTRCl:Y6/PNDIT-F3N/Ag	Initial	AM1.5G	13.26 ±3.6	0.81 ±0.112	62.69 ±13.15	25.37 ±0.43	1149.4 ±980.17	-58606.1 ±167257.52
	RB		15.73 ±0.31	0.853 ±0.003	72.6 ±0.66	25.39 ±0.57	60785.91 ±43369.8	-45.92 ±34.91
ITO/PEDOT:PSS/PM6:BTP-eC9/PDINN/Ag	Initial	AM1.5G	19.11 ±0.56	0.853 ±0.004	77.25 ±2.16	28.91 ±0.28	45801 ±59268	-5393.57 ±12199.55
	RB		19.21 ±0.29	0.855 ±0.004	77.71 ±0.27	28.81 ±0.35	654274 ±1105124	-12.06 ±10.59
	Initial	2600 K 1000 lux	19.63 ±9.8	0.571 ±0.246	57.05 ±17.41	170.04 ±3.42	45801 ±59268	-5393.57 ±12199.55
	RB		27.37 ±1.21	0.703 ±0.004	72.55 ±1.66	170.03 ±3.26	654274 ±1105124	-12.06 ±10.59
ITO/2PACz/PM6:L8BO (w DIB)/PNDIT-F3N/Ag	Initial	AM1.5G	14.14 ±7.28	0.717 ±0.346	64.36 ±21.23	24.68 ±3.95	926204 ±1674636	-235440.6 ±310006.49
	RB		18.66 ±0.18	0.89 ±0.004	78.33 ±0.49	26.76 ±0.43	1749952 ±2955609	-15.72 ±28.35

ITO/2PACz/PM6:L8BO (w/o additive)/PDINN/Ag	Initial	3000 K 1000 lux	14.44 ±12.46	0.470 ±0.334	44.52 ±28.92	160.18±13.96	710186 ±847908	-125.76 ±271.42
	RB		28.44 ±0.46	0.784 ±0.003	75.71 ±0.46	156.62±1.72	2066714 ±1321218	-0.88 ±0.59
ITO/ZnO/PM6:Y6/MoO_3/Ag	Initial	AM1.5G	15.81 ±1.31	0.829 ±0.006	72.21 ±5.53	26.3 ±0.37	12233 ±5812	-177.29 ±95.72
	RB		16.12 ±0.2	0.833 ±0.003	73.81 ±0.5	26.14 ±0.3	38789 ±18965	-40.03 ±18.14
	Initial	2600 K 1000 lux	12.07 ±5.77	0.519 ±0.211	38.29 ±8.76	175.61 ±5.06	12233 ±5812	-177.29 ±95.72
	RB		21.93 ±1.48	0.682 ±0.004	58.84 ±3.1	173.27 ±4.48	38789 ±18965	-40.03 ±18.14

Table S4 A summary of device performance for each subcell within an OPV module

Name	treat	R_s (Ω·cm^2)	R_{sh} (Ω·cm^2)	I_{leak} (μA)
subcell 1	Initial	2.13	6832	-18.0656
subcell 2		2.08	33700	-3.7272
subcell 3		1.96	53822	-1.6953
subcell 4		2.09	4	-14574.98
subcell 1	RB	2.11	1945147	-0.05602
subcell 2		2.07	3656307	-0.02451
subcell 3		1.95	2752925	-0.03582
subcell 4		1.88	6775297	-0.01502

Table S5 The photovoltaic performance of the homemade module before and after RB treatment under different light conditions.

Name	treat	Light	I_{SC} (μA)	V_{OC} (V)	FF (%)	P_{max} (μW)
ITO/2PACz/PM6:L8BO/PDINN/Ag	Initial	AM1.5G	1564.17±32.78	3.357±0.335	62.89±6.62	3329.68±610.68
		3000K 1000lux	11.62±0.55	1.828±0.87	46.32±9.35	10.74±5.8
	RB	AM1.5G	1541.89±35.76	3.592±0.044	70.94±2.03	3928.11±120.04
		3000K 1000lux	11.3±0.52	3.07±0.034	71.66±1.39	24.87±1.34
		3000K 200lux	2.46	2.818	66.63	4.62
		3000K 250lux	2.95	2.845	67.57	5.67
		3000K 500lux	6.36	2.958	69.43	13.06
		3000 K 1000 lux	11.52	3.035	72.23	25.25
		3000K 2000lux	22.34	3.109	74.08	51.46
		3000K 4000lux	41.85	3.181	75.83	100.95

Table S6 The minimal execution interval and average execution interval of the A-IoT sensor under different illuminances.

illuminance	minimal interval (s)	average interval (s)
200 lux	12.52	14.0434
250 lux	0.207	12.2132
500 lux	0.213	6.7883
1000 lux	0.206	3.4427
2000 lux	0.190	1.2521
4000 lux	0.164	0.5659

Table S7 Comparative overview of photovoltaic-powered wireless nodes

Year	Harvesting envelope			Communication metrics		Sticker-scale form and energy stack			Ref
	PV tech	Illumination range	PV size	Protocol[t]	Advertising interval	Per-area throughput[1] (Mbit·h^{-1}·cm^{-2})	Energy storage (rechargeable / battery-free)[2]	Form factor[3]	
2021	Monocrystalline silicon PV	3000 ~ 40000 lux	1.54 cm^2	N/A	N/A	N/A	N/A	front-end module	3
2022	Se PV	1000 lux	6.75 cm^2	RFID[p]	N/A	N/A	battery-free	panel	4
2022	OPV	Outdoor/Indoor (24h average luminance: 144 lux)	253 cm^2	Zigbee[a]	5/15/30 min according to light intensity	~ 0.000048	rechargeable (lithium polymer battery)	flexible module	5
2022	OPV	500 lux	40 cm^2	BLE[a]	N/A	N/A	rechargeable (supercapacitor)	panel	6
2023	InGaAs/GaAs/InGaP triple-junction PV	Outdoor	0.5 cm^2 or 1 cm^2	BLE[a]	20ms	N/A	rechargeable (supercapacitor, 7.5 mF + 100 μF)	microflier	7
2023	a copper(II/I) electrolyte based dye-sensitised PV	(1) factory: constant 1000 lux; (2) office: the illumination switched between 1000 lux and darkness; (3) home: natural light on top of artifcial lighting	22.4 cm^2	WiFi[a]	(1) 12.5min (2) 13min (3) 24.5min	~ 0.00006	rechargeable (supercapacitors, 2 × 1.5 F)	panel	8

2023	perovskite PV	(1) > 400 lux (2) 100 ~ 300 lux	(1) 2 cm^2 (2) 65.86 cm^2	BLE[a]	8000~60000 ms	～0.055	rechargeable (supercapacitor, mF-class)	wearable module	9
2024	perovskite PV	Indoor	1 cm^2	BLE[a]	5min	～0.003	N/A	panel	10
2024	Sb_2S_3 PV	1000lux	5 cm^2	BLE[a]	2000 ms	～0.089	rechargeable (supercapacitor)	panel	11
2024	Silicon PV	outdoor (Sunny/cloudy/Rainy)	dismater 75 mm	VLC[n]	30 min	N/A	rechargeable (Li-ion battery, 8 cells, total 200 mAh)	wearable module	12
2024	a-Si/a-SiGe/a-SiGe triple-junction PV	13 lux	320 cm^2	Backscatter[p,n]	N/A	N/A	rechargeable (supercapacitor)	flexible module	13
2024	OPV	one sun	6.25 cm^2	N/A	15min	N/A	rechargeable (Li-ion battery with NMC 622 Cathode)	panel	14
2025	perovskite PV	100-10000 lux	15 cm^2	Zigbee[a]	1000 ms	～0.073	rechargeable (supercapacitor, ~70 F)	sticker-scale patch	15

2025	Monocrystalline silicon PV	indoor	1.84 cm^2	N/A	N/A	N/A	rechargeable (supercapacitor, ~200 mF)	front-end module	16
2025	OPV	200-4000 lux	0.24 cm^2	BLE[a]	200 ms	18.6	battery-free (decoupling capacitor, 47 μF)	sticker-scale patch	this work

[t]Protocol superscripts: [a]Active = device transmits via its own radio (e.g., BLE, Zigbee); interoperable with commodity gateways and phones; aligns with this work (BLE[a]). [p]Passive = relies on external carrier/reader (e.g., Backscatter, RFID, VLC); deployment tied to instrumented environments. [n]Non-standard = proprietary or non-interoperable links.

[1]Per-area throughput = hourly net payload bits normalised by PV area; computed as payload per report × reports per hour/area. Continuous links may list peak link capacity. Used for relative comparison.

[2]Energy storage classification: “rechargeable” includes supercapacitors or secondary batteries; “battery-free” indicates no rechargeable storage beyond small decoupling capacitors.

[3]Form factor denotes device scale and integration (e.g., sticker-scale patch, wearable module, panel, flexible/stretchable).

Supplymentary Reference

1 Lee, N. J. *et al.* The interlayer screening effect of graphene sheets investigated by Kelvin probe force microscopy. *Appl. Phys. Lett.* **95** (2009). https://doi.org/10.1063/1.3269597

2 Fu, Y. *et al.* Molecular orientation-dependent energetic shifts in solution-processed non-fullerene acceptors and their impact on organic photovoltaic performance. *Nat. Commun.* **14**, 1870 (2023). https://doi.org/10.1038/s41467-023-37234-0

3 Yan, J. Z., Pan, W. H., Wu, H. H., Hsu, T. & Wei, C. L. Photovoltaic Energy Harvesting Chip With P&O Maximum Power Point Tracking Circuit and Novel Pulse-Based Multiplier. *IEEE Trans. Power Electron.* **36**, 12867–12876 (2021). https://doi.org/10.1109/TPEL.2021.3082533

4 Yan, B. *et al.* Indoor photovoltaics awaken the world's first solar cells. *Sci. Adv.* **8**, eadc9923 (2022). https://doi.org/doi:10.1126/sciadv.adc9923

5 Zhang, S. *et al.* Development of an organic photovoltaic energy harvesting system for wireless sensor networks; application to autonomous building information management systems and optimisation of OPV module sizes for future applications. *Sol. Energy Mater. Sol. Cells* **236**, 111550 (2022). https://doi.org/https://doi.org/10.1016/j.solmat.2021.111550

6 Jahandar, M., Kim, S., Kim, Y. H. & Lim, D. C. Large-Area Wide Bandgap Indoor Organic Photovoltaics for Self-Sustainable IoT Applications. *Adv. Energy Sustainability Res.* **4**, 2200117 (2023). https://doi.org/https://doi.org/10.1002/aesr.202200117

7 Johnson, K. *et al.* Solar-powered shape-changing origami microfliers. *Sci. Rob.* **8**, eadg4276 (2023). https://doi.org/doi:10.1126/scirobotics.adg4276

8 Michaels, H. *et al.* Emerging indoor photovoltaics for self-powered and self-aware IoT towards sustainable energy management. *Chem. Sci.* **14**, 5350–5360 (2023). https://doi.org/10.1039/D3SC00659J

9 Min, J. *et al.* An autonomous wearable biosensor powered by a perovskite solar cell. *Nat. Electron.* **6**, 630–641 (2023). https://doi.org/10.1038/s41928-023-00996-y

10 Pirc, M., Ajdič, Ž., Uršič, D., Jošt, M. & Topič, M. Indoor Energy Harvesting With Perovskite Solar Cells for IoT Applications─A Full Year Monitoring Study. *ACS Appl. Energy Mater.* **7**, 565–575 (2024). https://doi.org/10.1021/acsaem.3c02498

11 Chen, X. *et al.* Additive engineering for Sb2S3 indoor photovoltaics with efficiency exceeding 17%. *Light Sci. Appl.* **13**, 281 (2024). https://doi.org/10.1038/s41377-024-01620-0

12 Wang, S. *et al.* Sunflower-like self-sustainable plant-wearable sensing probe. *Sci. Adv.* **10**, eads1136 (2024). https://doi.org/doi:10.1126/sciadv.ads1136

13 Zhan, J.-L. *et al.* Flexible and wearable battery-free backscatter wireless communication system for colour imaging. *npj Flexible Electron.* **8**, 19 (2024). https://doi.org/10.1038/s41528-024-00304-4

14 Büttner, J. *et al.* Energy Harvesting and Storage with a High Voltage Organic Inorganic Photo-Battery for Internet of Things Applications. *Energy Technol.* **12**, 2301421 (2024). https://doi.org/https://doi.org/10.1002/ente.202301421

15 Han, W. *et al.* All Irradiance-Applicable, Perovskite Solar Cells-Powered Flexible Self-Sustaining Sensor Nodes for Wireless Internet-of-Things. *Adv. Funct. Mater.* **35**, 2425697 (2025). https://doi.org/https://doi.org/10.1002/adfm.202425697

16 Chakraborty, A. & Maity, A. A Battery-Less Energy Harvesting Front-End for Powering Multiple IoT Nodes Using Single Solar Cell: A System-Level Perspective. *IEEE Trans. Power Electron.* **40**, 14072–14083 (2025). https://doi.org/10.1109/TPEL.2025.3567569
