# Supplementary material for: Reverse-bias enabled mesoscale shunt passivation for organic photovoltaic modules to power miniaturised Ambient IoTs under low-light conditions
Source: Nat Commun. 2026 May 6;17:6109. doi: 10.1038/s41467-026-72623-1 (PMC13357597; doi:10.1038/s41467-026-72623-1)
Supplement: Supplementary file 3 — Transparent Peer Review file [file 41467_2026_72623_MOESM3_ESM.pdf]

nature portfolio

Peer Review File

Reverse-bias enabled mesoscale shunt passivation for organic photovoltaic modules to power miniaturised Ambient IoTs under low-light conditions

Corresponding Author: Professor Xinhui Lu

This manuscript has been previously reviewed at another journal. This document only contains information relating to versions considered at Nature Communications.

This file contains all reviewer reports in order by version, followed by all author rebuttals in order by version.

Version 0:

Reviewer comments:

Reviewer #1

(Remarks to the Author)
The authors have improved the manuscript massively according to all reviewers' comments, adding new data and explanation. With this in mind, I am satisfied with the current version to be accepted in Nature Communications.

Reviewer #4

(Remarks to the Author)
I have carefully examined the revised ms along with the previous reviewers reports and the authors detailed response. Based on this evaluation, I believe that the authors have addressed the main concerns raised during the earlier review process, particularly related to the novelty of the work.
The major criticisms in the previous review was that the observed effect might already be known and could be attributed to the shunt burning effect. In their response, the authors provide a thorough point by point clarification explaining why the phenomenon reported in this work cannot be attributed to shunt burning in state-of-the-art OPV devices. The revised manuscript now includes a clearer discussion of this aspect, which was previously missing. This addition significantly improves the clarity of the work.
The manuscript now presents a novel and important contribution from both a fundamental understanding and an application perspective. The authors identify the presence of mesoscale NFA agglomerations in the active layer that lead to variations in the large shunt resistance. According to me, this mechanism has not been previously discussed in the OPV literature. Furthermore, the authors provide sufficient experimental evidence to support their claims, including data indicating that the morphology remains largely unchanged upon implementing the this method, as reflected by the PL intensity measurements. Overall, I find that the authors have sufficiently addressed the reviewers concerns, and the novelty and broader implications of the work are now clearly conveyed in the revised ms.
I have one question for the authors that may be worth discussing. In temperature-dependent OPV measurements, it is often observed that the open-circuit voltage deviates from linear behavior at low temperatures and eventually turns downward. Historically, this behavior has been attributed to morphological effects or reduced charge generation. However, a later study https://doi.org/10.1002/solr.202000375 suggested that the phenomenon arises from high leakage current and demonstrated that the expected Voc behavior can be recovered by subtracting the lighted JV curve from the dark JV.
Given the mechanism proposed in the present work, it would be interesting to know whether a similar analysis could explain this long-standing observation in temperature-dependent measurements. In other words, could the methodology proposed here also help correct or avoid the low temperature Voc downturn observed in OPV devices?

Open Access This Peer Review File is licensed under a Creative Commons Attribution 4.0 International License, which permits use, sharing, adaptation, distribution and reproduction in any medium or format, as long as you give appropriate credit to the original author(s) and the source, provide a link to the Creative Commons license, and indicate if changes were made.
In cases where reviewers are anonymous, credit should be given to 'Anonymous Referee' and the source.
The images or other third party material in this Peer Review File are included in the article's Creative Commons license, unless indicated otherwise in a credit line to the material. If material is not included in the article's Creative Commons license and your intended use is not permitted by statutory regulation or exceeds the permitted use, you will need to obtain permission directly from the copyright holder.
To view a copy of this license, visit https://creativecommons.org/licenses/by/4.0/

Responses to comments from reviewers

We would like to thank the reviewers for their valuable time and comments, which have helped us significantly improve our paper's quality. We have addressed the comments from each reviewer point by point and revised the manuscript accordingly. Our detailed responses are shown in blue, and the corresponding revisions in both the manuscript and supplementary information are indicated with yellow highlights.

Reviewer #1 (Remarks to the Author):

The authors have improved the manuscript massively according to all reviewers' comments, adding new data and explanation. With this in mind, I am satisfied with the current version to be accepted in Nature Communications.

Response: We thank the reviewer #1 for the positive comments.

Reviewer #4 (Remarks to the Author):

I have carefully examined the revised ms along with the previous reviewers reports and the authors detailed response. Based on this evaluation, I believe that the authors have addressed the main concerns raised during the earlier review process, particularly related to the novelty of the work.

The major criticisms in the previous review was that the observed effect might already be known and could be attributed to the shunt burning effect. In their response, the authors provide a thorough point by point clarification explaning why the phenomenon reported in this work cannot be attributed to shunt burning in state-of-the-art OPV devices. The revised manuscript now includes a clearer discussion of this aspect, which was previously missing. This addition significantly improves the clarity of the work.

The manuscript now presents a novel and important contribution from both a fundamental understanding and an application perspective. The authors identify the presence of mesoscale NFA agglomerations in the active layer that lead to variations in the large shunt resistance. According to me, this mechanism has not been previously discussed in the OPV literature. Furthermore, the authors provide sufficient experimental evidence to support their claims, including data indicating that the morphology remains largely unchanged upon implementing the this method, as reflected by the PL intensity measurements.

Overall, I find that the authors have sufficiently addressed the reviewers concerns, and the novelty and broader implications of the work are now clearly conveyed in the revised ms.

I have one question for the authors that may be worth discussing. In temperature-dependent OPV measurements, it is often observed that the open-circuit voltage deviates from linear behavior at low temperatures and eventually turns downward. Historically, this behavior has been attributed to morphological effects or reduced charge generation. However, a later study https://doi.org/10.1002/solr.202000375 suggested that the phenomenon arises from high

leakage current and demonstrated that the expected Voc behavior can be recovered by subtracting the lighted JV curve from the dark JV.

Given the mechanism proposed in the present work, it would be interesting to know whether a similar analysis could explain this long-standing observation in temperature-dependent measurements. In other words, could the methodology proposed here also help correct or avoid the low-temperature Voc downturn observed in OPV devices?

Response: We thank Reviewer #4 for the positive comments and insightful suggestions. To understand the impact of leakage current on V_{OC} of OPVs at low temperature and the potential curing effect of our RB treatment, we have performed temperature (T)-dependent V_{OC} measurements on PM6-Y6 devices. The results have been added to **Figure S7**, and the details of the measurements have been included in the **Methods** section of the main text.

The initial device with a high leakage current shows an anomalous turnover in the V_{OC}–T plot at around 253 K (**Figure S7a** and **b**), consistent with the previous results [**Solar RRL, 2020, 4(11): 2000375.**]. Encouragingly, upon RB treating the same device at 253 K (**Figure S7c**), the leakage current is effectively reduced (**Figure S7d**), and the near-linear V_{OC}–T curve is recovered (**Figure S7a** and **b**). We note that, in this case, the RB treatment is performed at -5 V for 5 s, which differs from the RB treatment employed in the main text (-10 V for 15 s). This is because the V_{OC}–T is performed in air, with the device encapsulated using epoxy resin and cover glass, which reduces heat dissipation efficiency. Therefore, a lower RB with a shorter duration is employed to avoid overheating the device. To describe these results, we have added the following sentences to **line 129**, **page 5** of the main text.

"To further understand the roles of shunt pathways on key device metrics, we performed light-intensity (I)-dependent and temperature-dependent (T)-dependent J-V measurements on the same device before and after the RB treatment, as shown in **Figure 1i-l** and **Figure S7**. FF and V_{OC} drop significantly with decreasing light intensity in the initial device, as previously explained using a simple equivalent circuit model incorporating a finite shunt resistance.[8] In contrast, the V_{OC} of the RB-treated device strictly follows the ideal $kT/q\ln(I)$ dependence throughout the measured intensity range, while FF remains almost constant due to effective shunt passivation. On the other hand, the intensity-dependent J_{SC} curve shows no discernible difference before and after the RB treatment, consistent with the device statistics (**Figure 1h**) and results of external quantum efficiency (EQE) measurements (**Figure S8**). This is also consistent with our equivalent circuit modelling results, which indicate that J_{SC} is barely influenced by R_{sh} when it is larger than 100 $\Omega\cdot cm^2$ (**Figure S9**). Recent work has also suggested that high leakage current influences temperature (T)-dependent V_{OC} measurements, causing an anomalous turnover at low temperatures.[29] Encouragingly, we found that the RB treatment can also cure such behavior, restoring the expected near-linear V_{OC} increase with decreasing temperature (**Figure S7**). Overall, the alignment between device statistics, light-intensity-dependent, and temperature-dependent measurements clearly demonstrates the effectiveness of our RB treatment in enhancing the performance and reproducibility of OPV devices, particularly under low-light conditions. "

Our results indicate that the RB treatment can be performed before the V_{OC}-T measurements to eliminate the random shunting effect and thereby better reveal the intrinsic properties of the material system. To emphasise this point, we have modified the following sentences on **line 310** of **page 11** of the main text.

"Since light intensity-dependent and temperature-dependent *J-V* measurements that are widely applied to study the recombination kinetics and energetics of OPVs can be influenced by shunt pathways, the RB treatment is suggested to be performed before those measurements to eliminate the random shunting effect and thereby better reveal the intrinsic properties of the materials systems under investigation."

The results have been added to **Figure S7** in the Supplementary Information.

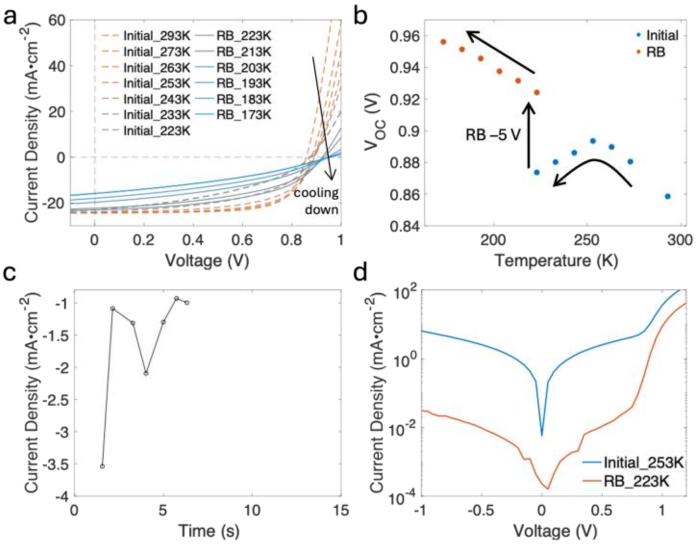

Figure S7 (a) Temperature-dependent light *J-V* curves of the same device before (dashed line) and after (solid line) the RB treatment. (b) V_{OC}-temperature plot of the same device before and after the RB treatment. (c) The real-time dark current density during the RB treatment at 223K. (d) The dark *J-V* curves of the same device before and after the RB treatment. The initial curve was measured at 253K, corresponding to the V_{OC} turnover temperature. The curve after the RB treatment was measured at 223K, the temperature at which the RB treatment was applied. In the temperature-dependent measurement, the device with low shunt resistance exhibits a turnover at 253 K. A reverse bias was subsequently applied at 223 K to eliminate the shunt pathway. Owing to inefficient heat dissipation in the encapsulated device, a relatively lower reverse bias (–5 V) with a shorter duration (5 s) was chosen to avoid device overheating. After the RB treatment, the leakage current is effectively suppressed, resulting in an increased V_{OC} at 223 K and the recovery of a linear relationship between V_{OC} and temperature.

We have added the following description on **line 367** of **page 13** of the main text.

"Temperature-dependent *J-V* measurement:

Current density–voltage characteristics were measured using an automatic photovoltaic efficiency measurement system equipped with a commercial solar simulator (LIV-1220, LightSky Technology Co., Ltd.). For temperature-dependent measurements, the devices were

mounted on a Linkam HFS600E-PB4 heating/freezing stage integrated into the optical path of the measurement system. The stage provides a temperature range from −196 to 600 °C with a temperature stability of 0.1 °C. During the measurements, the sample chamber was continuously purged with dry nitrogen to minimise moisture accumulation and ice condensation at low temperatures. At each target temperature, the stage was set to the desired value and held until thermal equilibrium was reached, as confirmed by a temperature fluctuation within ±0.1 °C, after which the *J-V* curve was recorded. All measurements were performed under a fixed one sun intensity condition."

Since Mr. Wenzhi Ma and Prof. Jun Yan carried out temperature-dependent measurements in response to Reviewer #4's comment, we have added them to the author list with full approval of all coauthors. The updated author list is as follows.

"Luhang Xu[1,*], Yuang Fu[1,*], Mianxin Xiao[2], Ho Ming Ng[3], Wenzhi Ma[4], Jun Yan[4], He Yan[3], Xin Li[5,2✉], Wei-Hsin Liao[2,6✉], Xinhui Lu[1✉]

[1] Department of Physics, The Chinese University of Hong Kong, Shatin, Hong Kong SAR 999077, China.
[2] Department of Mechanical and Automation Engineering, The Chinese University of Hong Kong, Shatin, Hong Kong SAR 999077, China.
[3] Department of Chemistry and Hong Kong Branch of Chinese National Engineering Research Centre for Tissue Restoration and Reconstruction, The Hong Kong University of Science and Technology, Clear Water Bay, Kowloon, Hong Kong SAR 999077, China.
[4] Guangdong Basic Research Centre of Excellence for Aggregate Science, School of Science and Engineering, The Chinese University of Hong Kong (Shenzhen), Shenzhen, Guangdong, 518172, China
[5] School of Civil Engineering, Harbin Institute of Technology, Harbin, China.
[6] Institute of Intelligent Design and Manufacturing, The Chinese University of Hong Kong, Shatin, Hong Kong SAR 999077, China.
[*] L. Xu and Y. Fu contributed equally to this work.
[✉] email: lixin01@hit.edu.cn; whliao@cuhk.edu.hk; xinhui.lu@cuhk.edu.hk"
